# Effectiveness and safety of Shenqi Fuzheng injection combined with platinum-based chemotherapy for treatment of advanced non-small cell lung cancer: a systematic review and meta-analysis

**DOI:** 10.3389/fonc.2023.1198768

**Published:** 2023-08-24

**Authors:** Chenxi Qiao, Shuaihang Hu, Dandan Wang, Kangdi Cao, Zhuo Wang, Xinyan Wang, Xiumei Ma, Zheng Li, Wei Hou

**Affiliations:** ^1^Department of Oncology, Guang’anmen Hospital, China Academy of Chinese Medical Sciences, Beijing, China; ^2^Graduate School of Beijing University of Chinese Medicine, Beijing, China

**Keywords:** non-small cell lung cancer, platinum-based chemotherapy, Shenqi Fuzheng injection, efficacy and safety, randomized controlled trial, systematic review, meta-analysis 2.2 retrieval strategy

## Abstract

**Objective:**

To evaluate the efficacy and safety of Shenqi Fuzheng Injection (SFI) combined with platinum-based chemotherapy (PBC) for the treatment of advanced non-small cell lung cancer (NSCLC).

**Methods:**

Seven electronic databases, including CNKI and Wanfang, were comprehensively searched to screen randomized controlled trials (RCTs) until May 1, 2022. The quality of each trial was evaluated according to the Cochrane Handbook for Systematic Reviews of Interventions, and systematic reviews were conducted according to the PRISMA guidelines. Statistical analysis was performed using Review Manager 5.3, and the results were expressed as relative risk (RR) and 95% confidence interval (95% CI). The primary outcome measures were objective response rate (ORR) and disease control rate (DCR). The secondary outcome measures were quality of life and toxicity. Subgroup analysis was performed according to the number of days of SFI single-cycle treatment and combined PBC regimen.

**Results:**

A total of 44 RCTs involving 3475 patients were included in the study. The meta-analysis results showed that, compared with PBC alone, SFI combined with PBC significantly improved the ORR (RR = 1.27, 95% CI = 1.18–1.37, P < 0.00001), DCR (RR = 1.12, 95% CI = 1.08–1.15, P < 0.00001), and quality of life (RR = 1.41, 95% CI = 1.31–1.52, P < 0.00001). It also reduced chemotherapy-induced hemoglobin reduction (RR = 0.57, 95% CI = 0.48–0.67, P < 0.00001), leukopenia (RR = 0.61, 95% CI = 0.53–0.71, P < 0.00001), thrombocytopenia (RR = 0.62, 95% CI = 0.55–0.70, P < 0.00001), and simple bone marrow suppression (RR = 0.55, 95% CI = 0.41–0.73, P < 0.0001). Nausea and vomiting (RR = 0.63, 95% CI = 0.52–0.77, P < 0.00001), diarrhea (RR = 0.48, 95% CI = 0.37–0.64, P < 0.00001), and simple digestive tract reactions (RR = 0.63, 95% CI = 0.49–0.80, P = 0.0002) also decreased with the treatment of SFI.

**Conclusion:**

SFI combined with PBC for the treatment of advanced NSCLC improved the ORR, DCR, and quality of life, and reduced the incidence of myelosuppression and gastrointestinal adverse reactions. However, considering the limitations of existing evidence, further verification using high-quality RCTs is required.

**Systematic review registration:**

https://inplasy.com/inplasy-2022-7-0026, identifier INPLASY202270026.

## 1 Introduction

GLOBOCAN 2020 data shows that lung cancer incidence and mortality are increasing annually worldwide (1). It is the most common type of malignant tumor and accounted for about 1.8 million deaths in 2020 (2). According to projections by the World Health Organization (WHO), by 2025 there may be 1 million people dying of lung cancer in China every year (3). The current incidence and mortality of lung cancer in China accounts for 37.0 and 39.8% of the world, respectively (1). Clinically, lung cancer is mainly divided into small cell lung cancer (SCLC) and non-small cell lung cancer (NSCLC). NSCLC accounts for approximately 85% of all lung cancers (4). The incidence of lung cancer in China is highest in the age group of 80–84-years (5). As the cancer onset is subtle, patients are often diagnosed in the middle and late stages, reducing the opportunity for surgical treatment and resulting in poor prognosis (6). For advanced patients with NSCLC without positive gene drive, platinum-based doublet chemotherapy is the first-line standard treatment (7), such as cisplatin or carboplatin with vinorelbine, paclitaxel, gemcitabine, or pemetrexed. However, the efficacy of chemotherapy is limited, and there are some disadvantages such as toxicity, side effects, reduced immunity, and high costs. In particular, the adverse bone marrow suppression and digestive system reactions affect the quality of life of patients, making it difficult for patients to complete the standard chemotherapy cycle. Therefore, reducing the side effects of chemotherapy, improving the immune function and quality of life of patients, and enhancing the effects of chemotherapy are urgent problems that need to be solved to prolong the survival of patients, making them current research hotspots.

In recent years, traditional Chinese medicine adjuvant chemotherapy has played an important role in the comprehensive treatment of lung cancer. Modern studies have shown that traditional Chinese medicine and its preparations use the broad-spectrum pharmacological effects of various components to affect multiple targets (8), regulate signaling pathways that mediate cancer cell invasion and metastasis, promote apoptosis, improve tumor microenvironment, and stimulate immune response to play an anti-NSCLC role (9, 10). A multicenter prospective cohort study by Zhang et al. (11) showed that traditional Chinese medicine can significantly prolong the disease-free survival of patients with NSCLC and reduce the non-hematologic toxicity of chemotherapy, especially nausea, loss of appetite, diarrhea, pain, and fatigue. Traditional Chinese medicine has the advantages of lower costs, toxicity, and side effects, individualized treatment based on syndrome differentiation, and good clinical tolerance. It can also alleviate some of the disadvantages of chemotherapy and has shown advantages as an adjuvant therapy.

Shenqi Fuzheng injection (SFI) (Limin Pharmaceutical Factory of Lizhu Group, Guangdong, China, Z19990065, China Food and Drug Administration (CFDA)) is a traditional Chinese medicine injection extracted using modern scientific techniques from the raw materials *Codonopsis pilosula* and *Astragalus membranaceus*. The effect of SFI is to strengthen the body and replenish qi. Studies have shown that SFI efficiently extended the overall survival by alleviating the oxidative stress injury in the animal model of amyotrophic lateral sclerosis, meanwhile the astragaloside IV, an active component of Radix Astragali significantly enhanced cell viability and suppressed apoptosis by increasing the expressions of Nrf2 and HO-1 (12), which might support the idea that SFI ‘strengthens the body’. It is widely used in the adjuvant treatment of colorectal, gastric, and breast cancers, as well as other advanced malignant tumors in China, and shows beneficial results (13–15). A number of clinical studies have reported that the combination of SFI and chemotherapy can improve the symptoms of lung and spleen qi deficiency and Karnofsky performance status (KPS) score in lung cancer, as well as reduce drug toxicity, alleviate adverse reactions of chemotherapy, improve the immune function and chemotherapy sensitivity of patients, delay tumor recurrence and metastasis, and have obvious advantages for short-term effectiveness (16).

At present, there are many clinical reports on SFI combined with platinum-based chemotherapy (PBC) in the treatment of NSCLC. However, most studies are low quality clinical trials, which failure to implement blinding, unscientific randomization methods, multiple confounding factors and risk of bias; the chemotherapy regimens are inconsistent, the short-term objective effective rate, toxicity, and side effects are different, and there are contradictory results.According to the Cochrane ‘RCT bias risk assessment tool’, each randomized controlled trial was evaluated for a separate risk of bias. The GRADE score was used to evaluate the level of evidence of all studies. The results showed that some studies had lower levels of evidence and higher risks. The results between the studies were quite different or even opposite. Therefore the quality of research is uneven. The efficacy of using SFI with PBC lacks support from large sample and multicenter clinical trials, limiting the value of the conclusions drawn. Leung et al. (17) reported that the combination of herbs or traditional Chinese medicine preparations with drugs may lead to various degrees of herb-drug interactions, which may be life-threatening. A real-world study by Wang et al. (18) showed that approximately 82.76% of SFI treatments in China were combined with chemical drugs, most of which inhibited gastric acid production and showed anti-tumor effects. It was also reported that the incidence of adverse drug reactions such as palpitation, chest tightness, chills, abdominal pain, dyspnea, and elevated blood pressure after injection of SFI was 0.17% (19). As the clinical efficacy of SFI has not yet reached an international consensus, this study used meta-analysis to conduct methodological analysis and quality evaluation by searching relevant national and international randomized controlled trials (RCTs) to provide medical evidence for the effectiveness and safety of SFI combined with PBC for the treatment of NSCLC, to guide clinical practice and further research.

## 2 Methods

### 2.1 Study design

This systematic review and meta-analysis strictly followed the preferred reporting items for systematic reviews and meta-analysis (PRISMA) guidelines (20). The registration number in the International Platform of Registered Systematic Review and Meta-analysis Protocols (INPLASY) is INPLASY202270026.

### 2.2 Retrieval strategy

Literature was sourced by searching PubMed, Cochrane Library, Embase, Web of Science, China National Knowledge Infrastructure, Wanfang Database, China Biomedical Database, and Chongqing VIP Chinese Science and Technology Periodical Full-text Database from inception to May 1, 2022. All relevant literature was searched to screen RCTs that included SFI combined with prescribed chemotherapy regimens. All literature was independently reviewed by two researchers (Suaihang Hu and Chenxi Qiao) to determine whether they met the inclusion criteria. Any disagreement arising in this process was resolved by consultation with a third researcher (Wei Hou).

The retrieval strategy of RCTs strictly followed the requirements of the Cochrane system evaluation manual, used the combination of subject words and free words for searching, and was adjusted according to the specific database. Multiple pre-searches were performed to determine the final retrieval strategy. Chinese search terms included: traditional Chinese medicine injection, Shenqi Fuzheng injection, Shenqi Fuzheng, lung cancer, and non-small cell lung cancer. English search terms included: lung cancer, non-small cell lung cancer, NSCLC, Chinese herbal injection, Chinese medicine injection, injection of TCM (traditional Chinese medicine), microemulsion injection, and Ginseng-Qi Fuzheng.

### 2.3 Inclusion and exclusion criteria

#### 2.3.1 Inclusion criteria

##### 2.3.1.1 Research type

RCTs of SFI combined with platinum-containing double-agent chemotherapy for the treatment of advanced NSCLC were published nationally and internationally, with or without blinding or allocation concealment. The language was limited to Chinese and English.

##### 2.3.1.2 Research object

Inclusion criteria was determined as follows: (1) Age was ≥ 18 years old and expected survival ≥ 3 months, with measurable clinical or observational indicators; (2) All cases were diagnosed as stage III–IV (according to WHO TNM staging) NSCLC by pathology or cytology, or were referred to as “advanced”; (3) Access was unrestricted to sex, race, nationality, economy, and education; (4) There were no contraindications related to chemotherapy or traditional Chinese medicine injection, no serious liver and kidney function, blood routine, and electrocardiogram abnormalities or other serious medical diseases, no obvious complications; (5) No patients received any concomitant radiotherapy, non-platinum chemotherapy, or other Chinese herbal medicine or Chinese patent medicine treatment, and there was non-postoperative or postoperative recurrence; (6) The baseline data of the two groups were similar and comparable.

##### 2.3.1.3 Intervention measures

The control group only received PBC treatment. The PBC regimen was defined as vinorelbine + cisplatin (NP), vinorelbine + carboplatin (NC), paclitaxel/albumin paclitaxel/paclitaxel liposome + cisplatin (TP), paclitaxel/albumin paclitaxel/paclitaxel liposome + carboplatin (TC), gemcitabine + cisplatin (GP), gemcitabine + carboplatin (GC), docetaxel + cisplatin (DP), docetaxel + carboplatin (DC), pemetrexed + cisplatin (AP), or pemetrexed + carboplatin (AC). The experimental group was treated with PBC combined with intravenous SFI. The dose and duration of the drugs used were not limited. According to the drug instructions of SFI, the standard dose of SFI is 250ml 1/day, and the dose range of SFI in this study is 200-260ml. In terms of the dose of chemotherapeutic drugs, gemcitabine was 1000-1500mg/m^2^, and the medication time was the 1st and 8th days of chemotherapy; vinorelbine was 25-40mg/m^2^, and the medication time was the 1st and 8th day of chemotherapy. Paclitaxel was 135-210mg/m^2^, and the medication time was the 1st day of chemotherapy. Pemetrexed was 510 mg/m^2^, and the medication time was the 1st day of chemotherapy. Cisplatin was 25-75mg/m^2^, and the medication time was the 1st to 3d days of chemotherapy, or 75-100mg/m^2^ was injected within one day; carboplatin was injected 300-500mg/m^2^ within one day.In each trial, the chemotherapy regimen was administered by intravenous drip.

##### 2.3.1.4 Outcome index

The outcome indexes were based on the WHO evaluation criteria for solid tumor efficacy (21) or Response evaluation criteria in solid tumors (RECIST) for solid tumor efficacy. These two methods have good consistency in the evaluation of tumor chemotherapy efficacy (22). WHO solid tumor efficacy evaluation criteria included: complete response (CR), complete disappearance of the tumor mass and duration of more than 1 month; partial response (PR), reduction of the product of tumor maximum diameter and maximum vertical diameter by 50% and maintained for more than 1 month; stable disease (SD), reduction in the product of the two diameters of the lesion by < 50% or increase by < 25% for more than 1 month; progressive disease (PD), increase in the product of the two diameters of the lesion by > 25% or appearance of new lesions. RECIST solid tumor efficacy evaluation criteria included: complete response (CR), tumor mass disappearance; partial response (PR), decrease in the tumor volume by more than 50% and normal auxiliary examination; stable disease (SD), decrease in the tumor volume by 50% or less and no improvement in auxiliary examination; progressive disease (PD), increase in the solid tumors by 25% or more and deterioration of the condition. The primary outcomes were objective response rate (ORR) and disease control rate (DCR). CR and PR were considered effective outcomes. Calculations were performed as follows: ORR = (CR + PR)/total number of cases; DCR = (CR + PR + SD)/total number of cases. The included studies contained the main outcome indicators.

Secondary outcome measures were quality of life improvement rate and incidence of adverse reactions (bone marrow suppression and gastrointestinal reactions). After the completion of the total course of treatment, the quality of life of patients was evaluated according to the KPS score: “improved score” was when the KPS score was improved >10 points, “stable score” when the KPS increased or decreased ≤10 points, and “decreased score” when KPS score decreased >10 points (23). Calculation of KPS improvement rate = (improved cases + stable cases)/total number of cases. Safety indicators were then assessed according to the WHO “acute and subacute toxicity criteria for chemotherapy drugs (24).” Bone marrow suppression was evaluated according to the occurrence of leukopenia, thrombocytopenia, and hemoglobin reduction. Gastrointestinal reactions were evaluated according to the occurrence of nausea, vomiting, and diarrhea. The incidence of adverse drug reactions is equal to the number of adverse reactions divided by the total number of cases. The included studies may or may not consist secondary outcome indicators or be evaluated with reference to other evaluation criteria.

#### 2.3.2 Exclusion criteria

The exclusion criteria included: (1) Non-RCTs or self-controlled studies, non-clinical trials such as case reports, experience summaries, cross-sectional studies or reviews, or those that did not implement real randomization or incorrectly established controls; (2) Patients with other primary tumors; (3) Intervention measures combined with radiotherapy, targeted surgery, other western medicine treatments, Chinese medicine compound, Chinese patent medicine, acupuncture, acupoint application, other Chinese medicine treatment, or SFI without chemotherapy; (4) SFI was administered non-intravenously; (5)Patients with severe complications such as serious hepatic and renal dysfunction, heart disease,diabetes, malnutrition, malignant anemia. (6) Lack of research on main outcome indicators; (7) The research data was incomplete or the data was wrong (such as obvious inconsistency in the number of cases before and after); (8) For repeatedly published literature, only publications of the highest quality, most recent year of publication, and with comprehensive information were selected following the quality evaluation of the literature; (9) Dissertations, abstracts, and other literature.

### 2.4 Data extraction

The retrieved studies were imported into NoteExpress software. Two researchers (Kangdi Cao and Zhuo Wang) browsed the topics, abstracts, and full texts according to the established inclusion and exclusion criteria, independently completed the screening and data extraction of the studies, and produced the flow chart. The relevant data from the final included studies were entered into an Excel table. The specific extraction contents included: (1) The first author, publication year, sampling and randomization methods, blind application, and other basic research information; (2) Sample size, age range, pathological type, disease stage and drug dose, and duration of the treatment group and the control group; (3) Outcome indicators, data, and evaluation scale; (4) The key factors of bias risk assessment of the study. When the relevant data was incomplete, the clinical trial leader was contacted by e-mail to supplement it. During literature screening and data extraction, the same standards and methods were adopted to reduce deviation. The results of the extracted data were compared and any disagreement was resolved by the third researcher (Wei Hou).

### 2.5 Methodological quality assessment

Two researchers (Shuaihang Hu and Chenxi Qiao) used the Cochrane Handbook for Systemic Review of Investments (version 5.1.0) RCT bias risk assessment tool to conduct a separate bias risk assessment for each RCT (25). The evaluation was carried out through the following seven contents: (1) whether the random sequence generation method was correct; (2) whether the allocation scheme hiding was described; (3) whether the researchers and subjects were blinded; (4) blind evaluation of research outcome; (5) integrity of outcome data; (6) whether to selectively report the research results; (7) other bias. The risk of bias in each field was evaluated as three levels: low risk, high risk, and unclear. Low risk level indicated that the test met all the criteria, whereas high risk indicated that any of the above items existed and the level of evidence was reduced. Unclear risk level indicated that it was neither high nor low risk, or the relevant content was not mentioned. The reasons for the evaluation level were recorded for high risk or unclear publications. The level of evidence for all studies was assessed by using GRADE (provided by the Cochrane Collaboration) (26). Any differences arising in this process were resolved through consultation with the third researcher (Wei Hou).

### 2.6 Statistical analysis

The Review Manager 5.3 software provided by the Cochrane Collaboration was used to generate forest maps using the included studies for meta-analysis. The data included were two categorical variables, and the effect value was expressed as relative risk (RR) and 95% confidence interval (95% CI). Differences were considered statistically significant when P < 0.05. The heterogeneity of included studies was analyzed using Cochran’s Q test and I^2^ test in Review Manager 5.3. When P > 0.1 and I^2^ < 50%, there was no significant heterogeneity in the included studies, and the fixed effect model(FEM) was used for combined analysis. When P < 0.1 and I^2^ > 50%, it was considered that there was significant heterogeneity in the included studies, and the source of heterogeneity was analyzed. Random effect model(REM) analysis was used when there was no clinical heterogeneity among the studies. Descriptive analysis was performed when there was significant clinical heterogeneity that disabled data combining.

### 2.7 Subgroup analysis

Subgroup analysis was performed according to the number of days of single-cycle SFI or the specific type of chemotherapy to reveal clinical heterogeneity and its effect on efficacy and safety. Studies using multiple chemotherapy regimens were not included in subgroup comparisons stratified by chemotherapy type.

### 2.8 Sensitivity analysis

Sensitivity analysis was carried out by limiting the literature to studies that met the “low deviation risk/high quality” criteria, such as excluding relevant studies with earlier publication years, smaller sample size, lower research quality, and insufficient or unclear allocation schemes. The impact on the overall effect size was observed to verify the robustness of the results; smaller influence was correlated with a more stable result. In other situations, the source of sensitivity was discussed. This paper excludes high-risk studies and studies published before 2010 to verify the stability of Meta-analysis results.

### 2.9 Publication bias

To ensure the reliability of the funnel plot assessment, we refer to Wang Shuo’s study (23). If at least ten included studies were available for meta-analysis, a funnel plot was drawn to assess potential publication bias by analyzing the distribution of the collected clinical data.

## 3 Literature screening results

### 3.1 Search process

**Figure 1:**
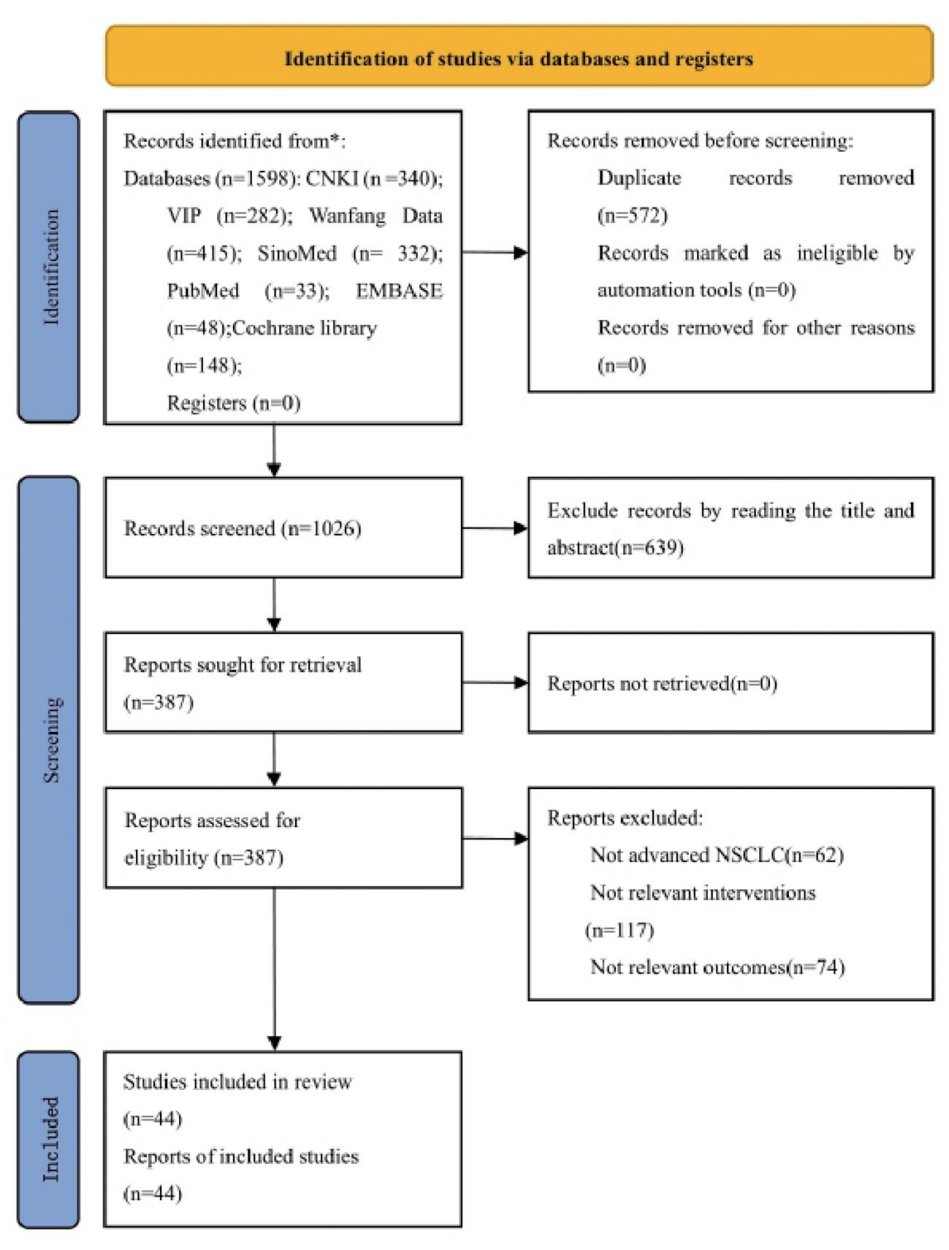
Flow chart of literature screening.

According to the defined search strategy, a total of 1598 articles were retrieved from the databases, including 340 articles from China National Knowledge Infrastructure (CNKI), 282 articles from VIP database, 415 articles from Wanfang database, 332 articles from China Biomedical Literature Service System (CBM), 33 articles from PubMed database, 148 articles from Cochrane Library database, and 48 articles from Embase database. After removing 572 duplicate articles, the titles and abstracts of the remaining 1026 articles were browsed. A total of 639 articles were removed that did not meet the inclusion criteria or were irrelevant. The full text was read of the remaining 387 articles, and a further 343 articles were excluded because they did not meet the criteria for advanced NSCLC, they used non-PBC regimens, no major outcome indicators were reported, data were incomplete, or they were non-RCT. The remaining 44 articles met the inclusion criteria (27–69). The literature screening process is detailed in **Figure 1**.

### 3.2 Characteristics of included studies

The RCTs included in this study were published between 2004–2021 and all were conducted in mainland China. In terms of the test population, a total of 3475 patients with advanced NSCLC were recruited, including 1745 in the experimental group and 1730 in the control group. Among them, one study (35) had incomplete outcome data. A total of 3460 patients had actual outcome data, including 1738 in the experimental group and 1722 in the control group. The number of males was 2216 and that of females was 1136, but the sum of the number of men and women in one study (48) was inconsistent with the total number of patients, and the number of biological sexin one study (40) was not recorded in detail. We contacted the author by email, but did not get a reply.The number of participants in each RCT ranged from 36–143. The age range was 25–83 years old.13 studies (27, 40, 48, 51, 53, 57, 60, 61, 64, 65, 67, 68, 70) only described the median age, and articles described the average age had a total of 2476 patients, with an average age of 62.21 years. 27 studies (27, 30, 33, 34, 36, 37, 39, 40, 43–45, 48, 50–53, 55, 56, 59, 61–68) included patients with KPS no less than 60 points, and 38 studies (27, 29–34, 36–39, 41–46, 48–53, 55–68, 70) included patients with expected survival of no less than 3 months. Five studies (27, 36, 45, 56, 62) carried out syndrome differentiation and only included people with qi deficiency. In terms of intervention measures, both the experimental group and the control group adopted the same PBC regimens: 13 studies (33–38, 40–46) adopted GP, 10 NP (49, 50, 52–59), 9 TP (61–69), 4 DP (30–33), 2 NC (48, 51), 1 AP (29), 1 GC (40), and 1 TC regimen (60). Three studies used a mixture of regimens: one used GP or TP (27), one used GP or AC (28), and one used TP, TC, or NP regimens (70). The experimental group was treated with intravenous infusion of SFI and the reported chemotherapy regimen. In terms of the evaluation indicators, all included studies reported ORR of short-term efficacy and DCR was reported or calculated, except for two studies (39, 63). Thirty-two studies (27, 29–31, 35, 36, 38, 39, 41–43, 45, 47–49, 51–61, 63, 64, 67–70) used WHO solid tumor efficacy criteria, eight studies (28, 32, 33, 37, 50, 62, 65, 66) used RECIST criteria, and four studies (34, 40, 44, 46) did not describe the efficacy criteria. A total of 25 studies (27, 30, 32, 33, 35–37, 39, 40, 47, 48, 50, 51, 53, 54, 56, 57, 60–62, 64, 66–69) evaluated the improvement in the quality of life by KPS score. Except for four studies (33, 44, 56, 58), all the included literature described the secondary outcome indicators with binary variables. For reporting adverse reactions, 26 studies (27, 29, 31, 32, 35–37, 40, 43, 47, 48, 50–52, 54, 55, 57, 60–68) adopted the performance and grading standards of acute and subacute adverse reactions of WHO, 2 studies (28, 59) adopted the grading standards of acute and subacute toxicity of anticancer drugs, and 16 studies (30, 33, 34, 38, 39, 41, 42, 44–46, 49, 53, 56, 58, 69, 70) did not explain the evaluation criteria. The number of incidence of bone marrow suppression was counted in 34 studies, of which 17 (27, 31, 35–37, 45, 47, 48, 50, 52, 57, 59, 64–68) reported hemoglobin reduction, 30 (27, 29, 31, 32, 34–37, 39, 41, 42, 45–47, 49–55, 57, 59, 61, 64–69) reported leukopenia, 27 (27, 31, 32, 34–37, 39, 41, 42, 45, 47–52, 54, 55, 57, 59, 61, 64–68) reported thrombocytopenia, and 3 (28, 30, 62) described only bone marrow suppression. The incidence of gastrointestinal reactions was described in 29 studies, of which 18 (29, 30, 35, 37, 46, 49–53, 55, 59–61, 64, 65, 68, 69) counted nausea and vomiting, 5 (29, 30, 35, 46, 51) counted diarrhea, and 11 (31, 32, 34, 39, 41, 42, 48, 57, 66, 67, 70) only described simple gastrointestinal reactions. The basic data of the included studies are shown in **Table 1**.

**Table 1:** Basic characteristics of the Included Studies.

Study ID	N(T/C)	Sex(M/F)	Age	TNM stages	Intervention group	Control group	Interested outcomes
Ding CJ 2012 (27)	35/35	42/28	38-70	IIIb-IV	250mL/day,10days/course;4 courses	GP/TP,4 courses	①②③④⑤⑥
Qi SG 2019 (28)	70/70	72/68	45-75	advanced	200mL/day;3 courses	GP/AC,3 courses	①②⑦
Ren JS 2015 (29)	42/42	49/35	53-73	IIIb-IV	250mL/day,10days/course;2 courses	AP,2 courses	①②⑤⑧⑨
Wang WM 2011 (30)	24/28	37/15	32-75	IV	250mL/day,10+ days;2 courses	DP,2 courses	①②③⑦⑧⑨
Yu F 2007 (31)	30/30	44/16	50-78	IIIb-IV	250mL/day,10days/course;2-3 courses	DP,2-3 courses	①②④⑤⑥⑩
Ma CG 2013 (32)	28/28	35/21	65-83	IIIa-IV	250mL/day,7days/course;3 courses	DP,3 courses	①②③⑤⑥⑩
Shan HG 2014 (33)	40/40	44/36	41-76	IIIb-IV	250mL/day,14days/course;2 courses	DP,2 courses	①②③
Bao Z 2019 (34)	47/47	61/33	65-71	advanced	250mL/day,21days/course;3 courses	GP,3 courses	①②⑤⑥⑩
Gui YX 2016 (35)	45/48	64/29	36-75	advanced	260mL/day,10days/course;4 courses	GP,4 courses	①②③④⑤⑥⑧⑨
Yao DJ 2013 (36)	50/50	84/16	30-70	III-IV	250mL/day,28days/course	GP,2 courses	①②③④⑤⑥
Zhao ZY 2014 (37)	50/52	80/22	49-67	IIIb-IV	250mL/day,10-14days/course	GP,2-6 courses	①②③④⑤⑥⑧
Zhang LM 2017 (38)	52/52	59/45	41-82	IIIb-IV	250mL/day,10days/course;2 courses	GP,2 courses	①②
Huang AX 2014 (39)	38/38	51/25	45-75	IIIb-IV	250mL/day,7days/course;2 courses	GP,2 courses	①②③⑤⑥⑩
Song Y 2007 (40)	59/58	UN	60-79	III-IV	250mL/day,10days/course;2 courses	GC,2 courses	①③
He WX 2021 (41)	48/48	58/38	56-78	III-IV	250mL/day,21days/course;4 courses	GP,4 courses	①②⑤⑥⑩
Jia J 2020 (42)	40/40	58/22	58-78	III-IV	UN	GP,4 courses	①②⑤⑥⑩
Li HT 2019 (43)	40/40	53/27	47-77	IIIb-IV	250mL/day,21days/course;2 courses	GP,2 courses	①②
Liu YF 2021 (44)	34/34	52/16	53-77	III-IV	250mL/day,10days/course;2 courses	GP,2 courses	①②
Luo BP 2018 (45)	48/48	61/35	33-64	IV	21days/course;2 courses	GP,2 courses	①②④⑤⑥
Wang HL 2021 (46)	53/53	58/48	47-73	IIIb-IV	250mL/day,21days/course;2 courses	GP,2 courses	①②⑤⑧⑨
Wu ZY 2019 (47)	28/28	29/27	38-71	advanced	250mL/day,21days/course;2 courses	GP,2 courses	①②③④⑤⑥
Wang YZ 2007 (48)	28/27	37/12	46-75	IIIb-IV	250mL/day,21days/course;3 courses	NC,3 courses	①②③④⑥⑩
Ding PQ 2016 (49)	60/60	78/42	62-80	III-IV	250mL/day,14days/course;2 courses	NP,2 courses	①②⑤⑥⑧
Wang TX 2014 (50)	41/41	60/22	43-80	III-IV	250mL/day,14days/course;2 courses	NP,2 courses	①②③④⑤⑥⑧
Jia YL 2012 (51)	72/71	98/45	60-77	IIIa-IV	250mL/day,14days/course;2 courses	NC,2 courses	①②③⑤⑥⑧⑨
Zhao ZY 2007 (52)	35/34	51/18	61-82	IIIa-IV	250mL/day,10days/course;2-3 courses	NP,2-3 courses	①②④⑤⑥⑧
Yu QZ 2007 (53)	30/32	65/19	35-76	III-IV	250mL/day,8-10days/course;4 courses	NP,4 courses	①②③⑤⑧
Wang K 2007 (54)	18/18	26/10	34-75	IIIb-IV	250mL/day,8days/course;3 courses	NP,3 courses	①②③⑤⑥
Li Y 2007 (55)	44/43	65/22	42-81	advanced	250mL/day,16days/course;4 courses	NP,4 courses	①②⑤⑥⑧
Geng L 2004 (56)	25/15	25/15	25-68	III-IV	250mL/day,21days/course;2 courses	NP,2 courses	①②③
Lv J 2008 (57)	40/40	65/15	51-78	advanced	250mL/day,21days/course;2 courses	NP,2 courses	①②③④⑤⑥⑩
Chen YF 2018 (58)	40/40	45/35	42-77	III-IV	250mL/day,14days/course;2 courses	NP,2 courses	①②
Zheng JH 2009 (59)	42/42	52/32	43-79	advanced	250mL/day,8days/course;3 courses	NP,3 courses	①②④⑤⑥⑧
Zou Y 2005 (60)	24/24	33/15	32-72	IIIa-IV	250mL/day,21days/course;2 courses	TC,2 courses	①②③⑧
Luo SZ 2006 (61)	25/25	33/17	33-75	IIIb-IV	250mL/day,21days/course;2 courses	TP,2 courses	①②③⑤⑥⑧
Cheng ZJ 2017 (62)	31/30	31/30	40-80	IIIb-IV	250mL/day,21days/course;2 courses	TP,2 courses	①②③⑦
Li HT 2012 (63)	30/30	44/16	49-82	IIIb-IV	250mL/day,10days/course;2 courses	TP,2 courses	①②
Luo SW 2007 (64)	30/30	39/21	33-75	IIIa-IV	250mL/day,14days/course;2 courses	TP,2 courses	①③④⑤⑥⑧
Liu R 2011 (65)	27/27	36/18	46-78	IIIa-IV	250mL/day,15days/course;2 courses	TP,2 courses	①②④⑤⑥⑧
Li DH 2014 (66)	50/40	57/33	38-74	IIIb-IV	250mL/day,14days/course;2 courses	TP,2 courses	①②③④⑤⑥⑩
Wang LY 2009 (67)	40/40	59/21	32-67	IIIa-IV	250mL/day,10-14days/course;2+ courses	TP,2+ courses	①②③④⑤⑥⑩
Zhang FL 2008 (68)	30/30	43/17	36-73	IIIa-IV	250mL/day,10-14days/course;2 courses	TP,2 courses	①②③④⑤⑥⑧
Zhao Q 2019 (69)	52/52	59/45	57-71	advanced	250mL/day,21days/course;2 courses	TP,2 courses	①②③⑤⑧
Wu L 2004 (70)	30/30	46/14	32-80	IIIb-IV	250mL/day,21days/course;2-3 courses	TP/TC/NP,2-3 courses	①②⑩

N, number of people; T/C, experimental group/control group; M/F, male/female; GP,gemcitabine + cisplatin; TP, paclitaxel/albumin paclitaxel/paclitaxel liposome + cisplatin; AC,pemetrexed + carboplatin; AP, pemetrexed + cisplatin; DP, docetaxel + cisplatin; GC,gemcitabine + carboplatin; NC, vinorelbine + carboplatin; NP, vinorelbine + cisplatin; TC, paclitaxel/albumin paclitaxel/paclitaxel liposome + carboplatin. ①Objective remission rate ORR=(CR+PR)/total cases×100%; ②Disease control rate DCR=(CR+PR+SD)/total cases×100%; ③KPS improvement rate=(number of improved cases + number of stable cases)/total cases; ④incidence of hemoglobin reduction = number of adverse reactions/total number of cases × 100%, calculated in the same way as below; ⑤incidence of leukopenia; ⑥incidence of thrombocytopenia; ⑦simple bone marrow suppression; ⑧incidence of nausea and vomiting; ⑨incidence of diarrhea; ⑩simple gastrointestinal reactions; UN, Unclear.

### 3.3 Methodological quality evaluation of included studies

**Figure 2:**
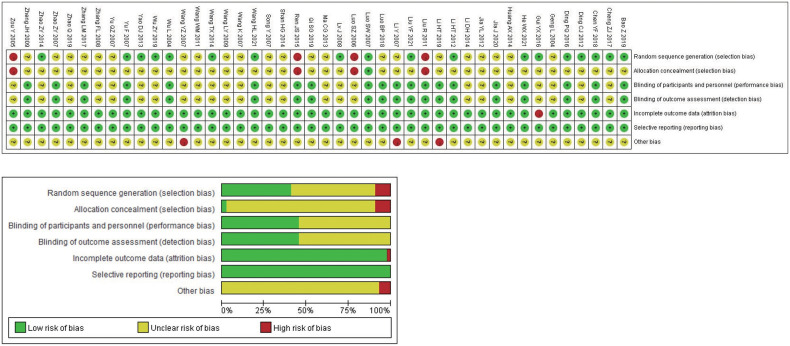
Methodological quality evaluation.

The Cochrane risk bias assessment tool was used to evaluate the methodological quality of the included studies. The 44 studies that met the inclusion criteria all described the baseline conditions, with no statistical difference. In terms of random sequence generation, all included studies mentioned random grouping, and 18 studies were evaluated as “low risk,” using either the random number table method (17 studies (27, 31, 34–37, 41, 44, 46, 47, 49, 50, 57, 58, 62, 63, 70)) or the envelope method (1 study (64)). Four studies (29, 60, 61, 65) were evaluated as “high risk” because the random method used the order of admission. Rest 22 studies (28, 30, 32, 33, 38–40, 42, 43, 45, 48, 51–56, 59, 66–69) did not describe the specific random method used, and there may be selective bias. In terms of allocation concealment and blindness, none of the included studies described concealment, no placebo was used, and no intention-to-treat analysis was performed; therefore, there may be selective and implementation bias. In blinding of researchers and subjects, blinding of outcome evaluators. All 44 studies had ORR primary objective indicators. In terms of subjective indicators, 19 studies (28, 29, 31, 34, 38, 41–46, 49, 52, 55, 58, 59, 63, 65, 70) did not have subjective indicators of KPS improvement rate and were evaluated as “low risk”. Although one study (64) analyzed the KPS improvement rate, it was still evaluated as “low risk” because the random method used was the envelope method and it was not subjectively affected. The results of 24 studies (27, 30, 32, 33, 35–37, 39, 40, 47, 48, 50, 51, 53, 54, 56, 57, 60–62, 66–69) included subjective indicators such as quality of life. It was difficult to estimate the impact on the results of the study, so the evaluation was “unclear” and there may be measurement bias. In terms of the integrity of the outcome data, some patients withdrew without a reported reason or ITT analysis in one study (35), resulting in a possibility of bias; the rest had no cases of withdrawal or loss of follow-up. The outcome indicators of all studies were fully reported without selective reporting bias. In terms of other sources of bias, the number of biological sex or pathological types in three studies (43, 48, 55) did not match the total number, which was evaluated as “high risk,” and there was no sufficient information to determine whether there were other sources of bias. The results of methodological quality evaluation are shown in **Figure 2**. The GRADE score is shown in **Table 2**, of which 6 are low-level evidence and 4 are very low-level evidence. The reasons for the downgrading are shown in the figure, indicating that the overall quality of the included literature was low and there were defects with respect to different aspects.

**Table 2:** GRADE score.

Quality assessment	No of RCTs	Design	Risk of bias	Inconsistency	Indirectness	Imprecision	Publication bias	RR(95% CI)	Quality
**ORR**	44	fixed trials	serious^1^	no serious	no serious	no serious	Strongly suspected^4^	1.27 (1.18–1.37)	⊕⊕◯◯ LOW
**DCR**	42	randomised trials	serious^1^	no serious	no serious	no serious	Strongly suspected^4^	1.12 (1.08–1.15)	⊕⊕◯◯ LOW
**KPS improvement**	25	randomised trials	Very serious^1^	serious^2^	no serious	no serious	Strongly suspected^4^	1.41 (1.31–1.52)	⊕◯◯◯ VERY LOW
**Hemoglobinia**	17	fixed trials	serious^1^	no serious	no serious	no serious	Strongly suspected^4^	0.57 (0.48–0.67)	⊕⊕◯◯ LOW
**Leukopenia**	30	randomised trials	serious^1^	serious^2^	no serious	no serious	Strongly suspected^4^	0.61 (0.53–0.71)	⊕◯◯◯ VERY LOW
**Thrombocytopenia**	27	fixed trials	serious^1^	no serious	no serious	no serious	Strongly suspected^4^	0.62 (0.55–0.70)	⊕⊕◯◯ LOW
**Myelosuppression**	3	fixed trials	serious^1^	no serious	no serious	Serious^3^	undetected	0.55 (0.41–0.73)	⊕⊕◯◯ LOW
**Nausea and Vomiting**	18	randomised trials	serious^1^	serious^2^	no serious	no serious	Strongly suspected^4^	0.63 (0.52–0.77)	⊕◯◯◯ VERY LOW
**Diarrhea**	5	fixed trials	serious^1^	no serious	no serious	Serious^3^	undetected	0.48 (0.37–0.64)	⊕⊕◯◯ LOW
**Gastrointestinal Reaction**	11	randomised trials	serious^1^	serious	no serious	Serious^3^	Strongly suspected^4^	0.63 (0.49–0.80)	⊕◯◯◯ VERY LOW

^1^ Unclear description of the hidden methods of random sequence and random allocation. ^2^ Point estimates vary widely from study to study. ^3^ The number of studies was too small and the confidence interval was too wide to be accurate.^4^ The funnel plots were asymmetrical, which indicated that publication bias might influence the results of the analysis.Objective remission rate ORR=(CR+PR)/total cases×100%; Disease control rate DCR=(CR+PR+SD)/total cases×100%; KPS improvement rate=(number of improved cases + number of stable cases)/total cases.

## 4 Meta-analysis results

### 4.1 SFI combined with PBC increases the objective response rate

All included studies reported ORR and had detailed data. Heterogeneity test analysis showed that there was no heterogeneity among the 44 studies (P = 0.98, I^2^ = 0%), so the FEM was used to combine the analysis. The results of meta-analysis showed that the ORR of the experimental group increased by approximately 27% compared with the control group (RR = 1.27, 95% CI = 1.18–1.37; combined effect test, Z = 6.42, P < 0.00001). This suggested that the ORR of the SFI + PBC group was significantly better than that of the PBC group.

**Figure 3:**
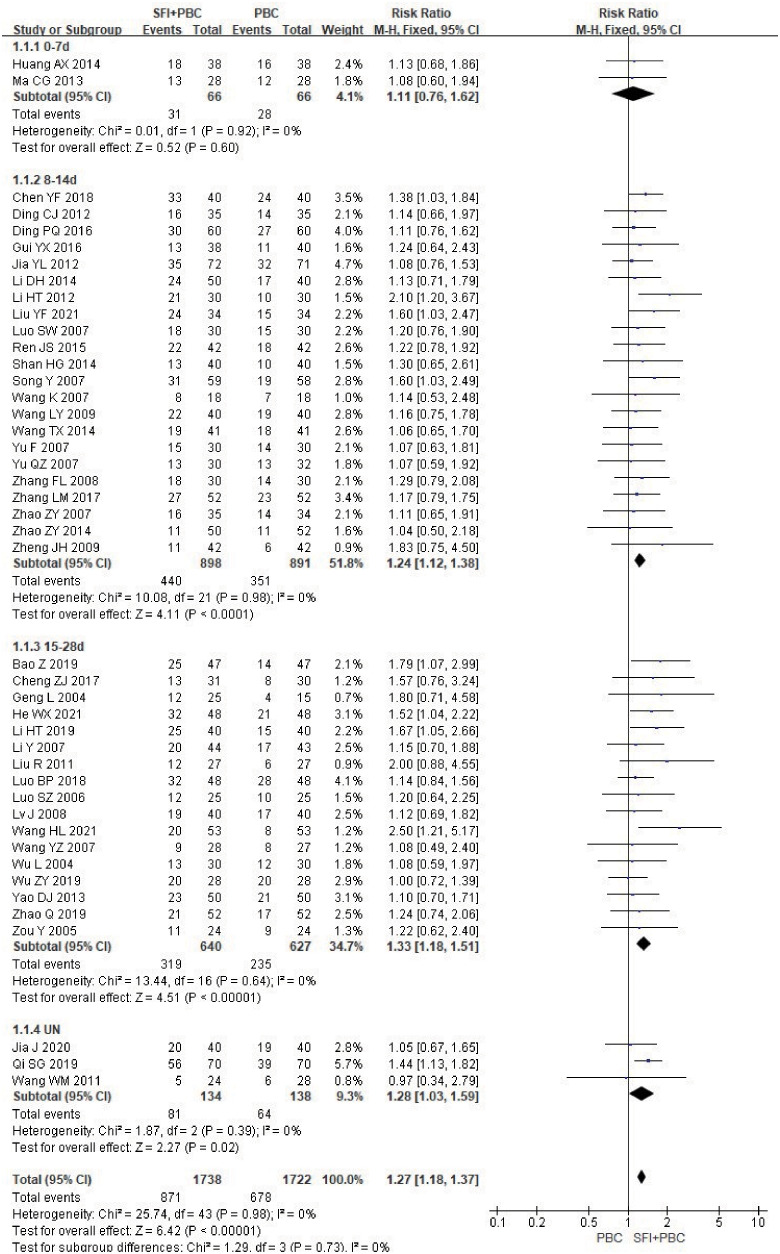
ORR forest plot stratified by days of single-cycle SFI dosing. Objective remission rate ORR=(CR+PR)/total cases×100%; UN, Unclear.

In the subgroup analysis of single-cycle SFI medication days, a total of 3460 patients were included, with 1738 in the experimental group and 1722 in the control group. There was no significant improvement in ORR when the single-cycle SFI medication was administered for 0–7 d (RR = 1.11, 95% CI = 0.76–1.62, P = 0.60, I^2^ = 0%) (**Figure 3**). However, significant improvements were observed in the 8–14 d group (RR = 1.24, 95% CI = 1.12–1.38, P < 0.0001, I^2^ = 0%) and 15–28 d group (RR = 1.33, 95% CI = 1.18–1.51, P < 0.00001, I^2^ = 0%). The results suggested that SFI + PBC had a significant advantage over PBC alone in improving ORR. While the longer single-cycle SFI medication days had the most obvious overall ORR improvement, this difference was not statistically significant (P = 0.73, I^2^ = 0%). Three studies (28, 30, 42) did not clearly explain the number of days of single-cycle SFI medication and the observation period. Meta-analysis showed that the ORR of the experimental group was better than that of the control group, and the effective rate was statistically significant (RR = 1.28, 95% CI = 1.03–1.59, Z = 2.27, P = 0.02), which was consistent with the original research results.

**Figure 4:**
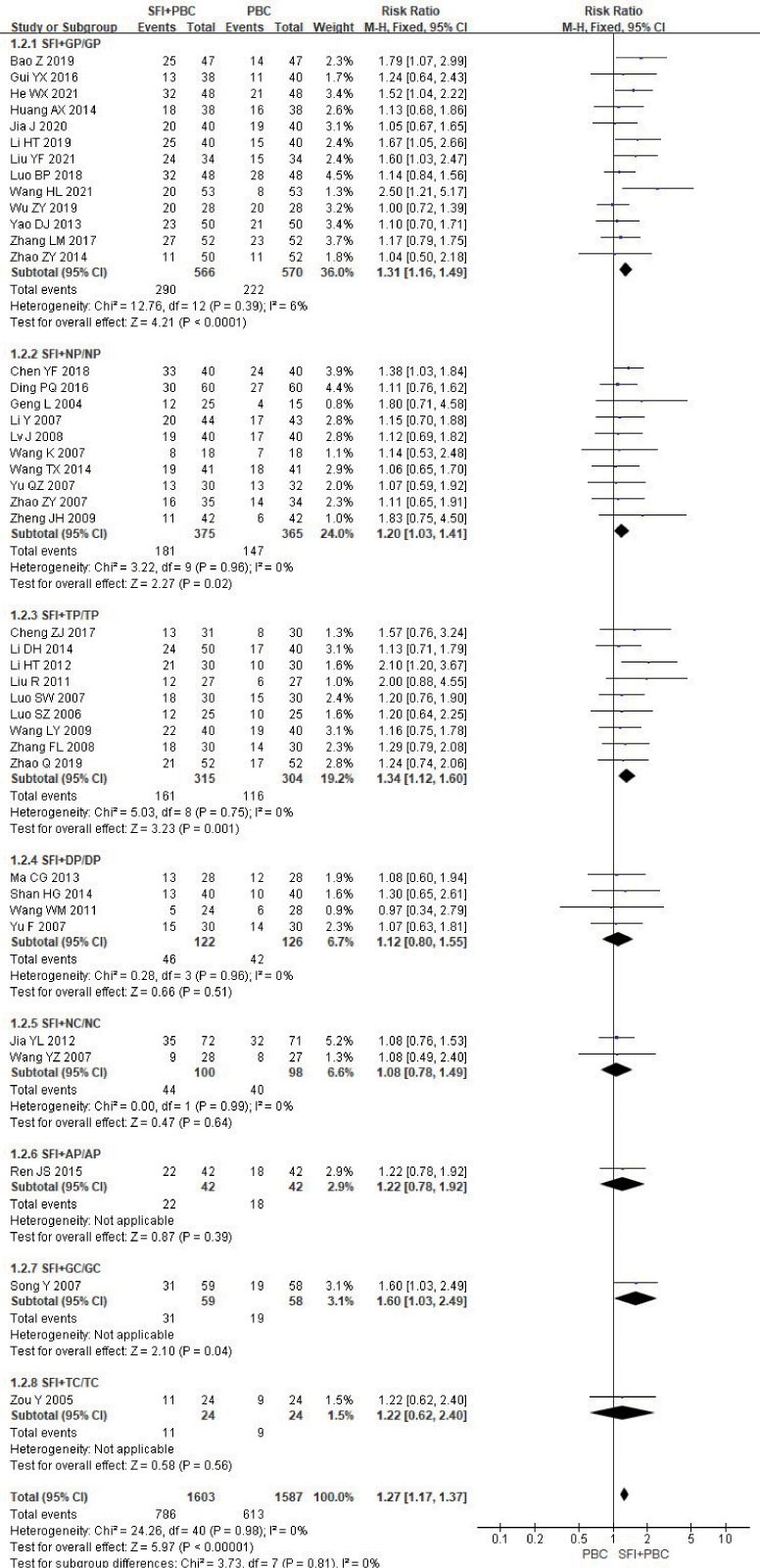
ORR forest plot stratified by chemotherapy regimen. Objective remission rate ORR=(CR+PR)/total cases×100%; GP, gemcitabine + cisplatin; NP, vinorelbine + cisplatin; TP, paclitaxel/albumin paclitaxel/paclitaxel liposome + cisplatin; DP, docetaxel + cisplatin; NC, vinorelbine + carboplatin; AP, pemetrexed + cisplatin; GC, gemcitabine + carboplatin; TC, paclitaxel/albumin paclitaxel/paclitaxel liposome + carboplatin.

In the stratified subgroup analysis of the combined specific chemotherapy type, 3190 patients were included after removing the three studies (27, 28, 70) using multiple chemotherapy regimens, with 1603 in the experimental group and 1587 in the control group. The ORR of SFI + GP (RR = 1.31, 95% CI = 1.16–1.49, P < 0.0001, I^2^ = 6%), SFI + NP (RR = 1.20, 95% CI = 1.03–1.41, P = 0.02, I^2^ = 0%), SFI + TP (RR = 1.34, 95% CI = 1.12–1.60, P = 0.001, I^2^ = 0%), and SFI + GC (RR = 1.60, 95% CI = 1.03–2.49, P = 0.04) for the treatment of NSCLC was significantly better than that of the PBC alone (**Figure 4**). However, no ORR improvement with SFI treatment compared with PBC alone was observed in SFI + DP (RR = 1.12, 95% CI = 0.80–1.55, P = 0.51, I^2^ = 0%), SFI + NC (RR = 1.08, 95% CI = 0.78–1.49, P = 0.64, I^2^ = 0%), SFI + AP (RR = 1.22, 95% CI = 0.78–1.92, P = 0.39), or SFI + TC (RR = 1.22, 95% CI = 0.62–2.40, P = 0.56) groups.

### 4.2 SFI combined with PBC increases the disease control rate

**Figure 5:**
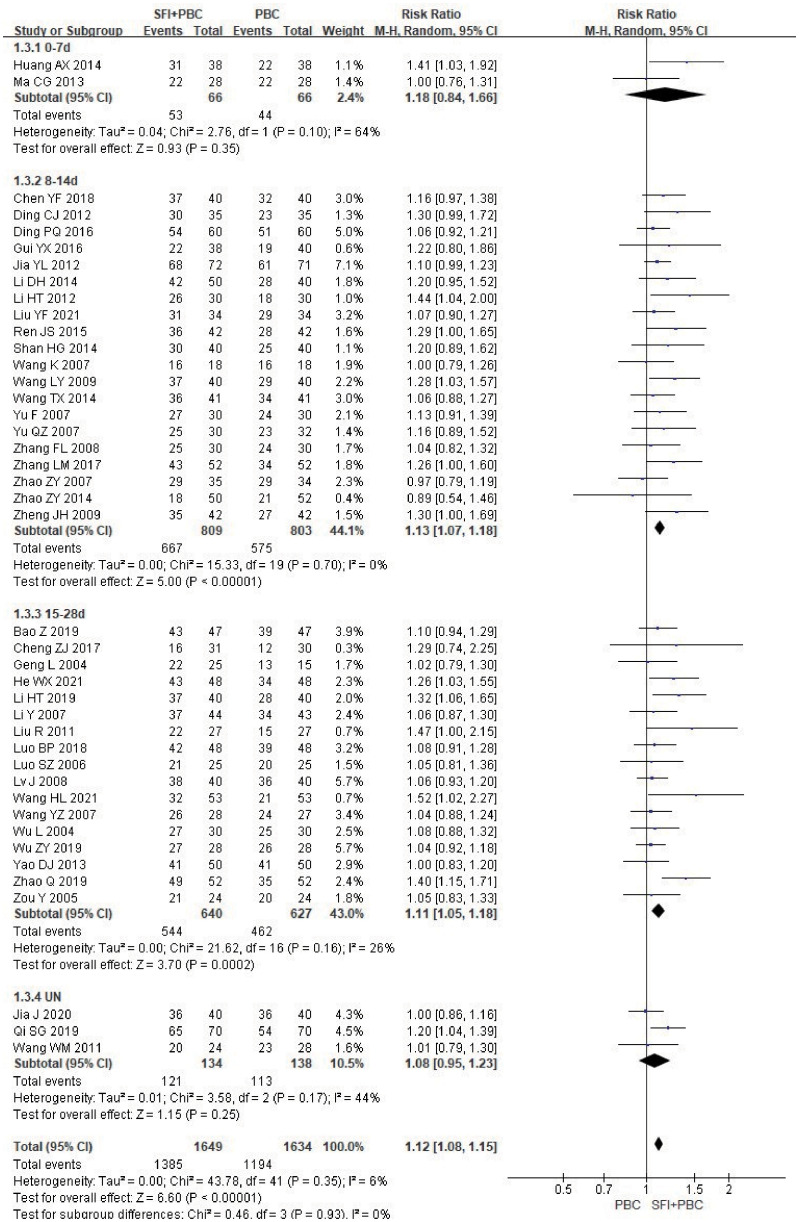
DCR forest plot stratified by days of single-cycle SFI dosing. Disease control rate DCR=(CR+PR+SD)/total cases×100%; UN, Unclear.

Only two articles (40, 64) did not report DCR, and statistical analysis of DCR could be performed for all other studies. In the subgroup analysis of single-cycle SFI medication days, a total of 3283 patients were included in the study, with 1649 in the experimental group and 1634 in the control group. Heterogeneity test analysis showed that there was heterogeneity in the 0–7 d subgroup (P = 0.10, I^2^ = 64%). However, because there were only two studies in this subgroup, further heterogeneity testing could not be performed. M-H method and REM were used for combined analysis. The results of meta-analysis showed that the use of SFI had little effect on the DCR when the number of days of single-cycle SFI was 0–7 d (RR = 1.18, 95% CI = 0.84–1.66, P = 0.35, I^2^ = 64%) (**Figure 5**). The DCR of the SFI + PBC group was significantly better than that of PBC alone group in 8–14 d (RR = 1.13, 95% CI = 1.07–1.18, P < 0.00001, I^2^ = 0%) and 15–28 d SFI treatment subgroups (RR = 1.11, 95% CI = 1.05–1.18, P = 0.0002, I^2^ = 26%). Overall combined analysis indicated that the DCR of SFI + PBC group was significantly better than that of PBC alone (RR = 1.12, 95% CI = 1.08–1.15, Z = 6.60, P < 0.00001). Three studies (28, 30, 42) did not clearly explain the number of days of single-cycle SFI medication and the observation period. Meta-analysis showed that the DCR of the experimental group was better than that of the control group, however, it was not statistically significant (RR = 1.08, 95% CI = 0.95–1.23, P = 0.25, I^2^ = 44%). There was no heterogeneity between the subgroups (P = 0.93, I^2^ = 0%), and the relationship between the number of days of single-cycle SFI medication and the DCR was not obvious.

**Figure 6:**
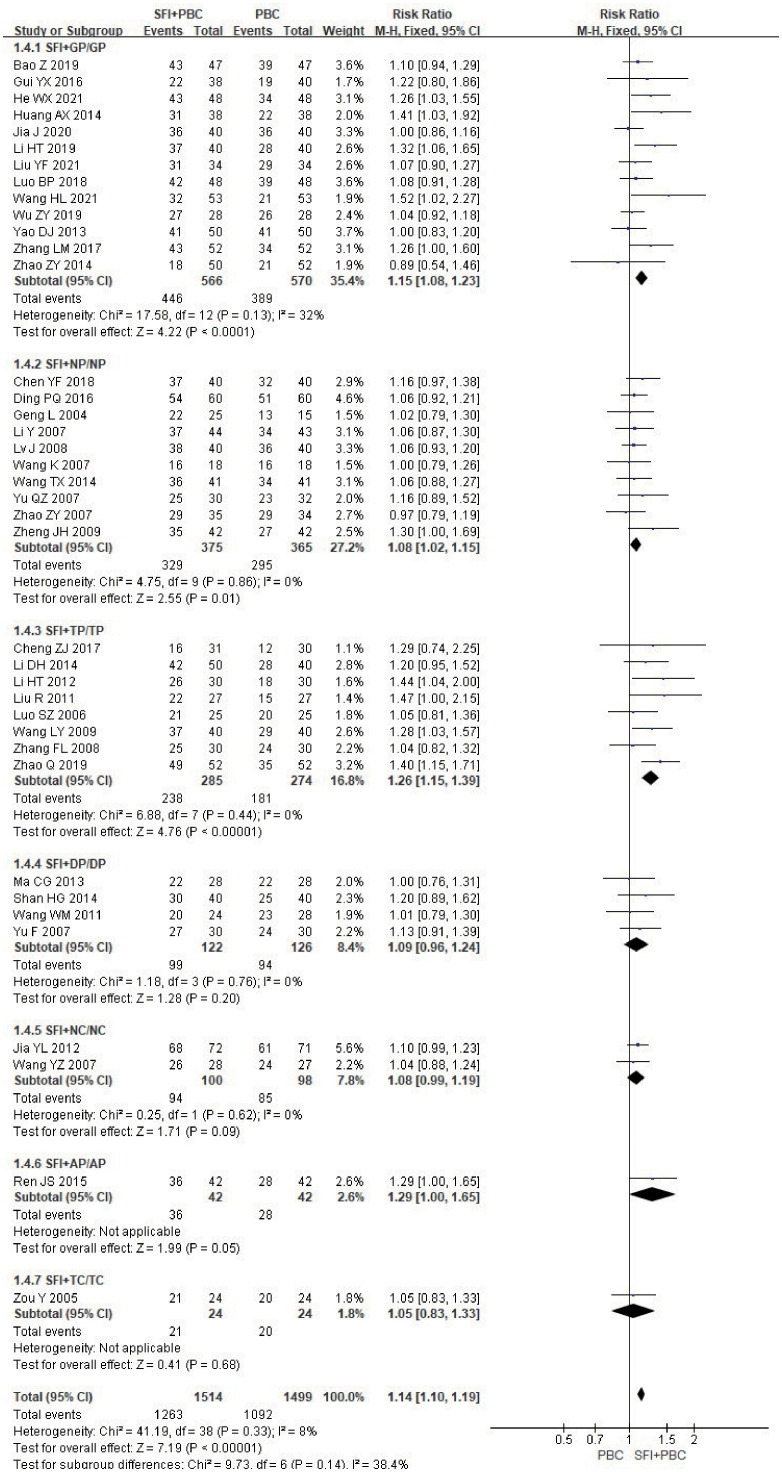
DCR forest plot stratified by chemotherapy regimen. Disease control rate DCR=(CR+PR+SD)/total cases×100%; GP, gemcitabine + cisplatin; NP, vinorelbine + cisplatin; TP, paclitaxel/albumin paclitaxel/paclitaxel liposome + cisplatin; DP, docetaxel + cisplatin; NC, vinorelbine + carboplatin; AP, pemetrexed + cisplatin; TC, paclitaxel/albumin paclitaxel/paclitaxel liposome + carboplatin.

In the subgroup analysis stratified by the specific type of chemotherapy combined, after removing 3 studies (27, 28, 70) using multiple regimens, a total of 3013 patients were included, with 1514 in the experimental group and 1499 in the control group. Heterogeneity test analysis showed that there was no heterogeneity in each group (P = 0.98, I^2^ = 0%), so M-H method and FEM were used for analysis. The subgroup results of SFI + GP (RR = 1.15, 95% CI = 1.08–1.23, P < 0.0001, I^2^ = 32%), SFI + NP (RR = 1.08, 95% CI = 1.02–1.15, P = 0.01, I^2^ = 0%), and SFI + TP (RR = 1.26, 95% CI = 1.15–1.39, P < 0.00001, I^2^ = 0%) suggested that SFI assisted GP, NP, TP chemotherapy could significantly improve the DCR, especially in the TP regimen (**Figure 6**). In contrast, SFI + DP (RR = 1.09, 95% CI = 0.96–1.24, P = 0.20, I^2^ = 0%), SFI + NC (RR = 1.08, 95% CI = 0.99–1.19, P = 0.09, I^2^ = %), SFI + AP (RR = 1.29, 95% CI = 1.00–1.65, P = 0.05), and SFI + TC (RR = 1.05, 95% CI = 0.83–1.33, P = 0.68) did not show any improvement; SFI assisted DP, NC, AP, and TC regimens had no improvement in DCR. However, only one study was included in the subgroups using AP and TC regimens, which may limit the accuracy of the conclusions. Overall, combined analysis showed that the DCR of the SFI + PBC group was significantly better than that of PBC alone (RR = 1.14, 95% CI = 1.10–1.19; combined effect size test Z = 7.19, P < 0.00001).

### 4.3 Quality of life

The KPS score was used to evaluate the quality of life. A total of 25 items (27, 30, 32, 33, 35–37, 39, 40, 47, 48, 50, 51, 53, 54, 56, 57, 60–62, 64, 66–69) were analyzed by two categorical variables. There was heterogeneity among the studies, so the REM was used to analyze the data. The results of meta-analysis showed that the improvement rate of KPS in the experimental group was approximately 41% higher than in the control group (RR = 1.41, 95% CI = 1.31–1.52; combined effect test Z = 8.93, P < 0.00001). This suggested that the improvement rate of KPS in the SFI + PBC group was significantly better than that in the PBC group.

**Figure 7:**
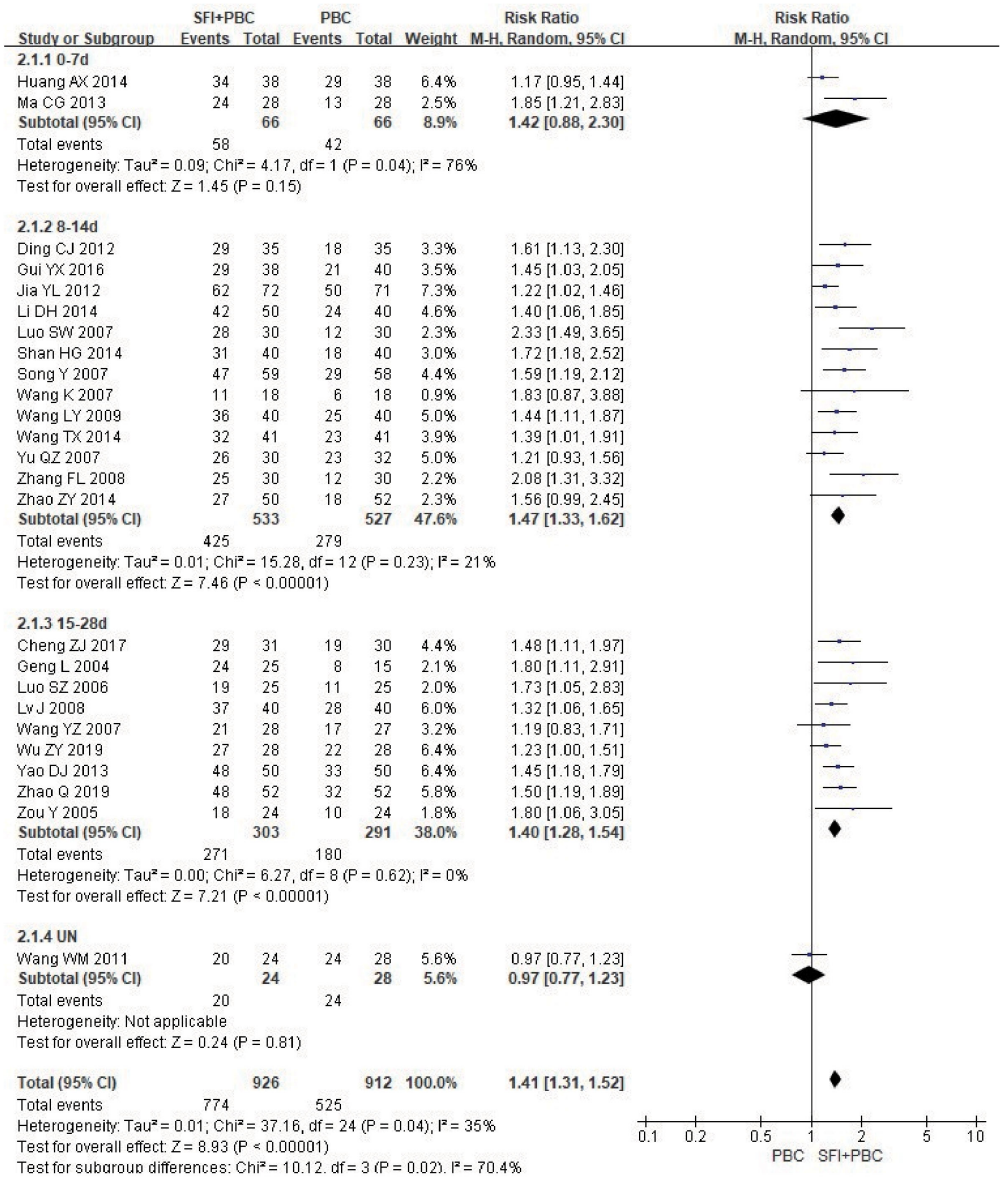
Forest plot of KPS improvement rate stratified by days of single-cycle SFI dosing. KPS improvement rate=(number of improved cases + number of stable cases)/total cases; UN, Unclear.

In the subgroup analysis of single-cycle SFI medication days, a total of 1838 patients were included, with 926 in the experimental group and 912 in the control group. Treatment with SFI in a single- cycle for 0–7 d (RR = 1.42, 95% CI = 0.88–2.30, Z = 1.45, P = 0.15) had no significant effect on KPS score improvement (**Figure 7**). The results of 8–14 d (RR = 1.47, 95% CI = 1.33–1.62, P < 0.00001, I^2^ = 21%) and 15–28 d (RR = 1.40, 95% CI = 1.28–1.54, P < 0.00001, I^2^ = 0%) subgroups showed that SFI treatment could effectively improve the quality of life. One study (30) did not specify the number of days of single-cycle SFI medication and the observation period. The results of meta-analysis showed that the KPS improvement rate of the experimental group was lower than that of the control group (RR = 0.97, 95% CI = 0.77–1.23, P = 0.81), which was inconsistent with the results of the original study. This discrepancy may be related to the fact that the included patients were all in the stage IV, and the quality of life was generally low and difficult to improve. There was heterogeneity between the groups (P = 0.02, I^2^ = 70.4%), suggesting that prolonging the duration of a single-cycle of SFI dosing had a significant improvement in the quality of life of the patients.

**Figure 8:**
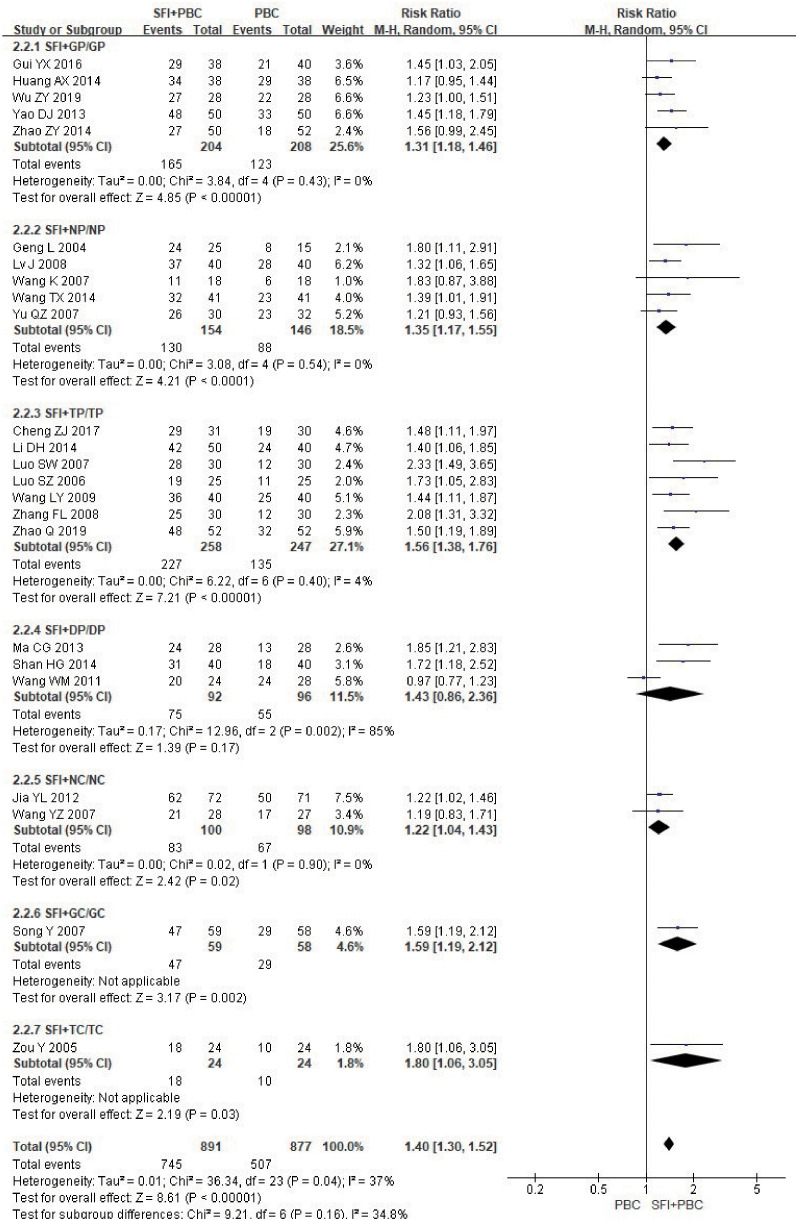
Forest plot of KPS improvement rate stratified by chemotherapy regimen. KPS improvement rate=(number of improved cases + number of stable cases)/total cases; GP, gemcitabine + cisplatin; NP, vinorelbine + cisplatin; TP, paclitaxel/albumin paclitaxel/paclitaxel liposome + cisplatin; DP, docetaxel + cisplatin; NC, vinorelbine + carboplatin; GC, gemcitabine + carboplatin;TC, paclitaxel/albumin paclitaxel/paclitaxel liposome + carboplatin.

In the subgroup analysis of the specific types of chemotherapy combined, one study (27) using multiple chemotherapy regimens was excluded. A total of 1768 patients were included, with 891 in the experimental group and 877 in the control group. The results showed that SFI combined with GP (RR = 1.31, 95% CI = 1.18–1.46, P < 0.00001, I^2^ = 0%), NP (RR = 1.35, 95% CI = 1.17–1.55, P < 0.0001, I^2^ = 0%), TP (RR = 1.56, 95% CI = 1.38–1.76, P < 0.00001, I^2^ = 4%), NC (RR = 1.22, 95% CI = 1.04–1.43, P = 0.02, I^2^ = 0%), GC (RR = 1.59, 95% CI = 1.19–2.12, P = 0.002), and TC regimens (RR = 1.80, 95% CI = 1.06–3.05, P = 0.03) significantly improved quality of life (**Figure 8**); SFI effectively improved the quality of life of patients with advanced NSCLC undergoing chemotherapy. In contrast, the results of SFI + DP (RR = 1.43, 95% CI = 0.86–2.36, P = 0.17, I^2^ = 85%) suggested that SFI had little significance in improving the quality of life of patients with DP chemotherapy, however, the heterogeneity of this group was high. Overall combined analysis showed SFI significantly improved the quality of life of chemotherapy patients (RR = 1.40, 95% CI = 1.30–1.52, P < 0.00001, I^2^ = 37%), and there was no significant difference between subgroups (P = 0.16, I^2^ = 34.8%).

Among the chemotherapy regimens, the SFI + DP subgroup did not pass the heterogeneity test, and the balance between the groups was poor. When the REM was selected, the combined RR was 1.43 (95% CI = 0.86–2.36). When the FEM was selected, the combined RR was 1.44 (95% CI = 1.18–1.76); changing the effect model had no obvious effect on the combined results. When the study by Wang et al. (30) (RR = 0.97, 95% CI = 0.77–1.23) was removed, I^2^ decreased to 0%, indicating that this study was the main source of heterogeneity. This may be related to the lack of included participants. The patients included were all in stage IV, with poor quality of life. After removal, there was a significant difference between the experimental group and the control group in this subgroup (RR = 1.78, 95% CI = 1.34–2.36, P < 0.0001), which was consistent with the original conclusion.

### 4.4 Bone marrow suppression

#### 4.4.1 Hemoglobin reduction

Seventeen studies (27, 31, 35–37, 45, 47, 48, 50, 52, 57, 59, 64–68) observed hemoglobin reduction events in 1276 patients, including 642 in the experimental group and 634 in the control group. Heterogeneity test analysis showed that there was no heterogeneity among the 17 studies (P = 0.78, I^2^ = 0%), so the FEM was used for analysis. The results showed that the red blood cell reduction rate in the experimental group was approximately 43% lower than in the control group (RR = 0.57, 95% CI = 0.48–0.67; combined effect size test Z = 6.63 and P < 0.00001). The incidence of hemoglobin reduction in the SFI + PBC group was significantly lower than in the PBC group.

Subgroup analysis based on the number of days of single-cycle SFI medication showed that when the single-cycle SFI medication was 8–14 d, the probability of hemoglobin reduction in SFI combined with PBC for NSCLC was 44% lower than with PBC alone (RR = 0.56, 95% CI = 0.46–0.69, P < 0.00001, I^2^ = 0%) (**Table 3**). Similar results were observed when the medication was administered for 15–28 d (RR = 0.58, 95% CI = 0.43–0.77, P = 0.0002, I^2^ = 0%). There was no heterogeneity among subgroups (P = 0.87, I^2^ = 0%).

**Table 3:** Analysis of toxicities and side effects stratified by days of single-cycle SFI dosing.

Subgroups	Number of studies	SFI+PBC n/N	PBC n/N	Heterogeneity	PooledRRs(95%CI)	Z	P
Hemoglobinia							
8-14d	11	94/421	163/414	P=0.76, I^2^ = 0%	0.56(0.46–0.69)	5.52	<0.00001
15-28d	6	51/221	88/220	P=0.43, I^2^ = 0%	0.58(0.43–0.77)	3.70	0.0002
Total	17	145/642	251/634	P=0.78, I^2^ = 0%	0.57(0.48–0.67)	6.63	<0.00001
Leukopenia							
0-7d	2	33/66	50/66	P=0.32, I^2^ = 0%	0.69(0.54–0.89)	2.91	0.004
8-14d	16	267/643	419/637	P<0.00001, I^2^ = 85%	0.62(0.49–0.78)	4.13	<0.0001
15-28d	11	160/462	275/461	P=0.15, I^2^ = 31%	0.59(0.50–0.71)	5.82	<0.00001
UN	1	15/40	25/40	Not applicable	0.60(0.38–0.96)	2.15	0.03
Total	30	475/1211	769/1204	P<0.00001, I^2^ = 77%	0.61(0.53–0.71)	6.49	<0.00001
Thrombocytopenia							
0-7d	2	13/66	30/66	P=0.56, I^2^ = 0%	0.43(0.25–0.75)	3.01	0.003
8-14d	14	170/571	250/563	P=0.23, I^2^ = 21%	0.67(0.58–0.78)	5.27	<0.00001
15-28d	10	101/385	167/383	P=0.13, I^2^ = 34%	0.60(0.49–0.74)	4.89	<0.00001
UN	1	11/40	22/40	Not applicable	0.50(0.28–0.89)	2.36	0.02
Total	27	295/1062	469/1052	P=0.12, I^2^ = 25%	0.62(0.55–0.70)	8.05	<0.00001
Myelosuppression							
Total	3	35/125	67/128	P=0.53, I^2^ = 0%	0.55(0.41–0.73)	4.01	<0.0001
Nausea and vomiting							
8-14d	12	195/494	307/502	P<0.0001, I^2^ = 71%	0.65(0.51–0.84)	3.28	0.001
15-28d	6	64/225	113/224	P=0.34, I^2^ = 11%	0.59(0.46–0.76)	4.01	<0.0001
Total	18	259/719	420/726	P=0.0002, I^2^ = 63%	0.63(0.52–0.77)	4.63	<0.00001
Diarrhea							
Total	5	46/229	96/234	P=0.11, I^2^ = 47%	0.48(0.37–0.64)	5.09	<0.00001
Gastrointestinal Reaction							
0-7d	2	40/66	52/66	P=0.02, I^2^ = 82%	0.71(0.36–1.43)	0.95	0.34
8-14d	3	38/120	65/110	P=0.12, I^2^ = 53%	0.56(0.36–0.88)	2.55	0.01
15-28d	5	82/193	129/192	P<0.0001, I^2^ = 85%	0.63(0.40–0.99)	2.02	0.04
UN	1	14/40	25/40	Not applicable	0.56(0.34–0.91)	2.34	0.02
Total	11	174/419	271/408	P<0.00001, I^2^ = 78%	0.63(0.49–0.80)	3.67	0.0002

n,number of cases with adverse reactions; N,total number of cases included in this study; UN,Unclear.

Subgroup analysis stratified by the specific type of chemotherapy combined was then performed. After removing one study (27) using multiple chemotherapy regimens, 1206 patients were included, with 607 in the experimental group and 599 in the control group. Compared with chemotherapy alone, SFI + GP (RR = 0.56, 95% CI = 0.42–0.76, P = 0.0002, I^2^ = 9%), SFI + NP (RR = 0.53, 95% CI = 0.37–0.74, P = 0.0003, I^2^ = 0%), SFI + TP (RR = 0.58, 95% CI = 0.40–0.83, P = 0.003, I^2^ = 12%), and SFI + DP groups (RR = 0.65, 95% CI = 0.43–0.98, P = 0.04) significantly reduced the incidence of hemoglobin reduction (**Table 4**). No advantage of SFI treatment was observed in the SFI + NC group (RR = 0.83, 95% CI = 0.32–2.15, P = 0.70), however, the number of included studies was small, and these results require further verification. There was no significant difference between the subgroups (P = 0.88, I^2^ = 0%).

**Table 4:** Toxic side effect analysis stratified by the specific type of chemotherapy combined.

Subgroups	Number of studies	SFI+PBC n/N	PBC n/N	Heterogeneity	Pooled RRs(95% CI)	Z	P
Hemoglobinia							
SFI+GP/GP	5	46/214	83/218	P=0.35, I^2^ = 9%	0.56(0.42–0.76)	3.77	0.0002
SFI+NP/NP	4	35/158	66/157	P=0.84, I^2^ = 0%	0.53(0.37–0.74)	3.65	0.0003
SFI+TP/TP	5	35/177	55/167	P=0.34, I^2^ = 12%	0.58(0.40–0.83)	2.96	0.003
SFI+DP/DP	1	15/30	23/30	Not applicable	0.65(0.43–0.98)	2.05	0.04
SFI+NC/NC	1	6/28	7/27	Not applicable	0.83(0.32–2.15)	0.39	0.70
Total	16	137/607	234/599	P=0.75, I^2^ = 0%	0.57(0.48–0.68)	6.29	<0.00001
Leukopenia							
SFI+GP/GP	10	158/440	255/444	P=0.17, I^2^ = 30%	0.64(0.54–0.76)	5.05	<0.00001
SFI+NP/NP	8	141/310	215/310	P<0.00001, I^2^ = 91%	0.66(0.47–0.95)	2.27	0.02
SFI+TP/TP	7	87/254	162/244	P=0.11, I^2^ = 42%	0.52(0.40–0.68)	4.90	<0.00001
SFI+DP/DP	2	26/58	41/58	P=0.41, I^2^ = 0%	0.66(0.48–0.90)	2.64	0.008
SFI+NC/NC	1	40/72	52/71	Not applicable	0.76(0.59–0.97)	2.17	0.03
SFI+AP/AP	1	9/42	17/42	Not applicable	0.53(0.27–1.05)	1.82	0.07
Total	29	461/1176	742/1169	P<0.00001, I^2^ = 77%	0.61(0.53–0.71)	6.29	<0.00001
Thrombocytopenia							
SFI+GP/GP	9	109/387	169/391	P=0.02, I^2^ = 58%	0.63(0.46–0.87)	2.81	0.005
SFI+NP/NP	7	81/280	126/278	P=0.59, I^2^ = 0%	0.67(0.54–0.82)	3.79	0.0001
SFI+TP/TP	6	40/202	84/192	P=0.88, I^2^ = 0%	0.45(0.33–0.62)	4.97	<0.00001
SFI+DP/DP	2	18/58	27/58	P=0.22, I^2^ = 33%	0.67(0.37–1.20)	1.34	0.18
SFI+NC/NC	2	32/100	47/98	P=0.80, I^2^ = 0%	0.67(0.48–0.93)	2.39	0.02
Total	26	280/1027	453/1017	P=0.14, I^2^ = 23%	0.62(0.54–0.72)	6.65	<0.00001
Myelosuppression							
Total	3	35/125	67/128	P=0.53, I^2^ = 0%	0.55(0.41–0.73)	4.01	<0.0001
Nausea and vomiting							
SFI+GP/GP	3	40/141	70/145	P=0.02, I^2^ = 73%	0.53(0.24–1.19)	1.54	0.12
SFI+NP/NP	6	102/252	138/252	P=0.01, I^2^ = 65%	0.75(0.54–1.04)	1.74	0.08
SFI+TP/TP	5	46/164	111/164	P=0.002, I^2^ = 77%	0.40(0.22–0.72)	3.02	0.003
SFI+DP/DP	1	13/24	15/28	Not applicable	1.01(0.61–1.67)	0.04	0.97
SFI+NC/NC	1	35/72	49/71	Not applicable	0.70(0.53–0.94)	2.42	0.02
SFI+AP/AP	1	18/42	30/42	Not applicable	0.60(0.40–0.89)	0.01	0.01
SFI+TC/TC	1	5/24	7/24	Not applicable	0.71(0.26–1.94)	0.66	0.51
Total	18	259/719	420/726	P=0.0002, I^2^ = 63%	0.63(0.52–0.77)	4.63	<0.00001
Diarrhea							
Total	5	46/229	96/234	P=0.11, I^2^ = 47%	0.48(0.37–0.64)	5.09	<0.00001
Gastrointestinal Reaction							
SFI+GP/GP	4	78/173	120/173	P=0.004, I^2^ = 78%	0.64(0.43–0.96)	2.17	0.03
SFI+NP/NP	1	13/40	22/40	Not applicable	0.59(0.35–1.00)	1.96	0.05
SFI+TP/TP	2	23/90	47/80	P=0.81, I^2^ = 0%	0.45(0.30–0.66)	4.04	<0.0001
SFI+DP/DP	2	25/58	38/58	P=0.16, I^2^ = 49%	0.66(0.40–1.09)	1.62	0.10
SFI+NC/NC	1	25/28	25/27	Not applicable	0.96(0.82–1.14)	0.43	0.67
Total	10	164/389	252/378	P<0.00001, I^2^ = 79%	0.64(0.49–0.82)	3.40	0.0007

NP, vinorelbine + cisplatin; NC, vinorelbine + carboplatin; TP, paclitaxel/albumin paclitaxel/paclitaxel liposome + cisplatin; TC ,paclitaxel/albumin paclitaxel/paclitaxel liposome + carboplatin; GP, gemcitabine + cisplatin; GC, gemcitabine + carboplatin; DP, docetaxel + cisplatin; DC, docetaxel + carboplatin; AP, pemetrexed + cisplatin; AC, pemetrexed + carboplatin.

#### 4.4.2 Leukopenia

Thirty studies (27, 29, 31, 32, 34–37, 39, 41, 42, 45–47, 49–55, 57, 59, 61, 64–69) used dichotomous variables to report the reduction of white blood cells, with detailed data for a total of 2415 patients, including 1211 in the experimental group and 1204 in the control group. Heterogeneity test analysis showed that there was significant heterogeneity among the included studies (P < 0.00001, I^2^ = 77%), so the REM was used. The results of pooled analysis showed that the rate of leukopenia in the experimental group was approximately 39% lower than in the control group (RR = 0.61, 95% CI = 0.53–0.71; combined effect size test Z = 6.49, P < 0.00001), suggesting that the use of SFI with PBC helped to reduce the occurrence of leukopenia.

In the subgroup analysis stratified by the number of days of single-cycle SFI medication, treatment for 0–7 d (RR = 0.69, 95% CI = 0.54–0.89, P = 0.004, I^2^ = 0%), 8–14 d (RR = 0.62, 95% CI = 0.49–0.78, P < 0.0001, I^2^ = 85%), and 15–28 d (RR = 0.59, 95% CI = 0.50–0.71, P < 0.00001, I^2^ = 31%) significantly reduced the incidence of leukopenia. While there was a correlation between increasing the number of days of single-cycle SFI medication and RR of leukopenia improvement, the difference between the groups was not statistically significant (P = 0.81, I^2^ = 0%). One study (42) did not describe the medication time. The results of meta-analysis showed that the incidence of leukopenia in the SFI + PBC group was lower than in the PBC group (RR = 0.60, 95% CI = 0.38–0.96, P = 0.03). The difference between the two groups was statistically significant, which was consistent with the original conclusion.

In the subgroup analysis of the specific types of chemotherapy combined, after removing one study (27) using multiple chemotherapy regimens, 2345 patients were included, with 1176 in the experimental group and 1169 in the control group. The results showed SFI + GP (RR = 0.64, 95% CI = 0.54–0.76, P < 0.00001, I^2^ = 30%), SFI + NP (RR = 0.66, 95% CI = 0.47–0.95, P = 0.02, I^2^ = 91%), SFI + TP (RR = 0.52, 95% CI = 0.40–0.68, P < 0.00001, I^2^ = 42%), SFI + DP (RR = 0.66, 95% CI = 0.48–0.90, P = 0.008, I^2^ = 0%), and SFI + NC (RR = 0.76, 95% CI = 0.59–0.97, P = 0.03) could significantly reduce white blood cells compared with PBC alone. The greatest improvement was observed in the SFI + TP group, however, the difference between the groups was not significant (P = 0.47, I^2^ = 0%). SFI + AP (RR = 0.53, 95% CI = 0.27–1.05, P = 0.07) did not significantly improve leukopenia, but only 1 study was included in this subgroup, so further research is required to draw accurate conclusions.

Heterogeneity test analysis showed that there was significant heterogeneity in the subgroup of 8–14 d of SFI single-cycle (P < 0.00001, I^2^ = 85%), subgroup of chemotherapy with the NP regimen (P < 0.00001, I^2^ = 91%), and overall combined analysis (P < 0.00001, I^2^ = 77%). After excluding individual studies one by one, it was found that after removing Wang (54), the I^2^ of the subgroup with 8–14 d of SFI single-cycle was reduced to 48%, the subgroup with NP regimen was reduced to 57%, and the overall combined I^2^ was reduced to 37%. After removing the studies of Wang (54) and Zheng (59) individually and at the same time, I^2^ decreased to 30%, 31% and 31%, respectively, indicating that these two studies were the main sources of heterogeneity. This may be because the sample size used by Wang (54) was small, with 18 patients in the experimental and control group having leukopenia, and the cisplatin dosage by Zheng (59) small (25 mg/m^2^) compared to other studies and bone marrow suppression was weak. The results after eliminating these studies were consistent with the original analysis (RR = 0.63, 95% CI = 0.58–0.70, P < 0.00001, I^2^ = 31%).

#### 4.4.3 Thrombocytopenia

A total of 27 studies (27, 31, 32, 34–37, 39, 41, 42, 45, 47–52, 54, 55, 57, 59, 61, 64–68) observed thrombocytopenia events with detailed data for 2114 patients, including 1062 in the experimental group and 1052 in the control group. Heterogeneity test analysis showed that there was no significant difference between the 27 studies (P = 0.12, I^2^ = 25%), hence, the FEM was used. The overall analysis results showed that the incidence of thrombocytopenia in the experimental group was approximately 38% lower than in the control group (RR = 0.62, 95% CI = 0.55–0.70; combined effect size test Z = 8.05, P < 0.00001); the SFI + PBC group reduced the incidence of thrombocytopenia during the treatment of advanced NSCLC compared with the PBC group.

Subgroup analysis based on the number of days of single-cycle SFI medication showed that treatment for 0–7 d (RR = 0.43, 95% CI = 0.25–0.75, P = 0.003, I^2^ = 0%), 8–14 d (RR = 0.67, 95% CI = 0.58–0.78, P < 0.00001, I^2^ = 21%), and 15–28 d (RR = 0.60, 95% CI = 0.49–0.74, P < 0.00001, I^2^ = 34%) could significantly improve the occurrence of thrombocytopenia. However, there was no significant correlation between the degree of improvement and the duration of single-cycle SFI (P = 0.36, I^2^ = 6.6%). One study (42) did not report the number of days of medication. The incidence of thrombocytopenia in the experimental group was significantly lower than that in the control group (RR = 0.50, 95% CI = 0.28–0.89, P = 0.02), which was consistent with the original conclusion.

In the subgroup analysis of the specific types of chemotherapy combined, one study (27) using multiple chemotherapy regimens was excluded. A total of 2044 patients were included, with 1027 in the experimental group and 1017 in the control group. Due to the large heterogeneity within the SFI + GP group (P = 0.02, I^2^ = 58%), a REM was used. Results of SFI + GP (RR = 0.63, 95% CI = 0.46– 0.87, P = 0.005, I^2^ = 58%), SFI + NP (RR = 0.67, 95% CI = 0.54–0.82, P = 0.0001, I^2^ = 0%), SFI + TP (RR = 0.45, 95% CI = 0.33–0.62, P < 0.00001, I^2^ = 0%), and SFI + NC (RR = 0.67, 95% CI = 0.48–0.93, P = 0.02) suggested that SFI combined with PBC significantly improved thrombocytopenia. There was no significant difference between the groups (P = 0.33, I^2^ = 13%). There was also no significant alleviation of thrombocytopenia in patients with the SFI + DP regimen (RR = 0.67, 95% CI = 0.37–1.20, P = 0.18, I^2^ = 33%).

The SFI + GP subgroup did not pass the heterogeneity test (P = 0.02, I^2^ = 58%). After excluding three studies (34, 37, 45), I^2^ decreased to 0%, indicating that these three articles were the main source of heterogeneity. In the Bao study (34), heterogeneity may have been introduced because the patients included were too old (over 65 years old), and the hematopoietic function of bone marrow was easily restricted, resulting in slow platelet production, or may have been related to taking anti-platelet and blood-activating drugs at the same time. In the Zhao study (37), the dosage of cisplatin was high (80–100 mg/m^2^), and many cycles were used to evaluate the efficiency; most other studies observed 2 cycles to evaluate the efficacy, whereas they study observed 2–6 cycles and patients may have stopped treatment because they could not tolerate the continued treatment. In the Luo study (45), patients included were in stage IV, most of the basic hematopoietic levels were poor, and the SFI dosage was not specified. The results after exclusion of these studies were consistent with the original conclusion.

#### 4.4.4 Simple bone marrow suppression

Three studies (28, 30, 62) only described simple bone marrow suppression and did not specify the specific type of bone marrow suppression. These studies included a total of 253 patients, with 125 cases in the experimental group and 128 cases in the control group. Heterogeneity test analysis showed that there was no heterogeneity among the three studies (P = 0.53, I^2 = ^0%), so the FEM was used for combined analysis. The results showed that the incidence of simple bone marrow suppression in the experimental group was approximately 45% lower than that in the control group (RR = 0.55, 95% CI = 0.41–0.73; combined effect size test Z = 4.01, P < 0.0001), indicating that SFI combined with PBC could significantly improve the incidence of simple bone marrow suppression.

### 4.5 Digestive tract reaction

#### 4.5.1 Nausea and vomiting

Eighteen studies (29, 30, 35, 37, 49–53, 55, 59–61, 64, 65, 68, 69) observed the occurrence of nausea and vomiting with detailed data, including a total of 1445 patients, with 719 in the experimental group and 726 in the control group. Heterogeneity test analysis showed that there was significant heterogeneity among the 18 studies (P = 0.0002, I^2^ = 63%), so the REM was used for analysis. The overall analysis results showed that the incidence of nausea and vomiting in the experimental group was approximately 37% lower than that in the control group (RR = 0.63, 95% CI = 0.52–0.77; combined effect size test Z = 4.63, P < 0.00001). Therefore, the incidence of nausea and vomiting in the SFI + PBC group was significantly lower than that in the PBC group.

In the subgroup analysis of single-cycle SFI medication days, treatment for 8–14 d (RR = 0.65, 95% CI = 0.51–0.84, P = 0.001, I^2^ = 71%) and 15–28 d (RR = 0.59, 95% CI = 0.46–0.76, P < 0.0001, I^2^ = 11%) could reduce the incidence of nausea and vomiting, and there was no significant difference between the groups (P = 0.58, I^2^ = 0%).

In the subgroup analysis stratified by the specific type of chemotherapy, SFI + TP (RR = 0.40, 95% CI = 0.22–0.72, P = 0.003, I^2^ = 77%), SFI + NC (RR = 0.70, 95% CI = 0.53–0.94, P = 0.02), and SFI + AP (RR = 0.60, 95% CI = 0.40–0.89, P = 0.01) subgroups had a significant effect on reducing nausea and vomiting in patients compared with TP, NC, and AP chemotherapy alone. The subgroup results of SFI + GP (RR = 0.53, 95% CI = 0.24–1.19, P = 0.12, I^2^ = 73%), SFI + NP (RR = 0.75, 95% CI = 0.54–1.04, P = 0.08, I^2^ = 65%), SFI + DP (RR = 1.01, 95% CI = 0.61–1.67, P = 0.97), and SFI + TC (RR = 0.71, 95% CI = 0.26–1.94, P = 0.51) showed that SFI had no advantage in reducing the incidence of nausea and vomiting compared with GP, NP, DP, and TC chemotherapy regimens.

Four subgroups did not pass the heterogeneity test (overall combined P = 0.0002, I^2^ = 63%): the 8–14 d SFI single-cycle subgroup (P < 0.0001, I^2^ = 71%) and GP (P = 0.02, I^2 = ^73%), NP (P = 0.01, I^2^ = 65%), and TP (P = 0.002, I^2^ = 77%) chemotherapy subgroups. After removing four studies (46, 59, 64, 68), the overall combined I^2^ decreased to 0%, indicating that these four studies were the main source of heterogeneity. In the study performed by Wang (46), the heterogeneity may have been because the range of KPS scores of the enrolled patients was not described. In the Zheng (59) study, the cisplatin dosage was smaller than other studies (25 mg/m^2^) and the occurrence of nausea and vomiting treatment group/control group was 15/4, indicating there may have been a data entry error. Finally, Luo (64) and Zhang (68) were the only two studies to use the TP chemotherapy regimen. The heterogeneity in this subgroup may have been derived from the different pathological types of the included patients. After excluding these two studies, the conclusion was consistent with the original analysis (RR = 0.70, 95% CI = 0.62–0.78, P < 0.00001, I^2^ = 0%).

#### 4.5.2 Diarrhea

Five studies (29, 30, 35, 46, 51) reported the incidence of diarrhea with detailed data on a total of 463 patients, including 229 in the experimental group and 234 in the control group. Heterogeneity test analysis showed that there was no significant heterogeneity among the five studies (P = 0.11, I^2^ = 47%), hence, the FEM was used for combined analysis. The results of meta-analysis showed that the incidence of diarrhea in the experimental group was approximately 52% lower than in the control group (RR = 0.48, 95% CI = 0.37–0.64; combined effect size test Z = 5.09, P < 0.00001). Therefore, the incidence of diarrhea in SFI + PBC treatment of advanced NSCLC was lower than that of PBC alone.

#### 4.5.3 Simple gastrointestinal reaction

Eleven studies (31, 32, 34, 39, 41, 42, 48, 57, 66, 67, 70) observed the occurrence of simple gastrointestinal reactions and had detailed data for a total of 827 patients, including 419 in the experimental group and 408 in the control group. Heterogeneity test analysis showed that there was significant heterogeneity among the 11 studies (P < 0.00001, I^2^ = 78%), so the REM was used for combined analysis. The overall results showed that the incidence of simple gastrointestinal reactions in the experimental group was approximately 37% lower than in the control group (RR = 0.63, 95% CI = 0.49–0.80; combined effect size test Z = 3.67, P = 0.0002), indicating that the incidence of simple gastrointestinal reactions in SFI + PBC was significantly lower than that in PBC alone. However, the heterogeneity within the subgroup was large, with few studies included in the analysis, limiting the credibility of the conclusion.

In the subgroup analysis stratified by the number of days of single-cycle SFI medication, no significant improvement of simple gastrointestinal reactions was observed in the 0–7 d group (RR = 0.71, 95% CI = 0.36–1.43, P = 0.34, I^2^ = 82%). Whereas, a significant improvement was observed in the 8–14 d (RR = 0.56, 95% CI = 0.36–0.88, P = 0.01, I^2^ = 53%) and 15–28 d (RR = 0.63, 95% CI = 0.40–0.99, P = 0.04, I^2^ = 85%) subgroups. This suggested that prolonging the days of medication significantly improved the simple gastrointestinal reaction.

In the subgroup analysis of the specific chemotherapy types combined, one study (70) with multiple chemotherapy regimens was excluded. A total of 767 patients were included, with 389 in the experimental group and 378 in the control group. SFI + GP (RR = 0.64, 95% CI = 0.43–0.96, P = 0.03, I^2^ = 78%) and SFI + TP (RR = 0.45, 95% CI = 0.30–0.66, P < 0.0001, I^2^ = 0%) showed significant differences between the experimental group and the control group. However, no significant difference was observed with SFI + NP (RR = 0.59, 95% CI = 0.35–1.00, P = 0.05), SFI + DP (RR = 0.66, 95% CI = 0.40–1.09, P = 0.10, I^2^ = 49%), and SFI + NC (RR = 0.96, 95% CI = 0.82–1.14, P = 0.67), suggesting that SFI had little effect on the simple digestive tract reaction of NP, DP, and NC chemotherapy. Only one study was included that used either SFI + NP or SFI + NC, which may have affected the accuracy of the conclusion.

Due to the heterogeneity among the 11 studies, individual studies were excluded one by one for re-analysis. Three studies were identified as the main sources of heterogeneity: removing Yu (31) decreased the I^2^ of the 8–14 d SFI single-cycle medication subgroup from 57% to 0%; removing Wang (48) decreased the I^2^ of the 15–18 d subgroup from 85% to 0%; removing Huang (39) decreased the I^2^ of the SFI + GP subgroup from 78% to 0%; and removing both Huang and Wang decreased the overall I^2^ from 78% to 0%. The heterogeneity introduced by Yu (31) may have been because patients were included with KPS scores less than 60, which was lower than other groups and easier to impact digestive tract reaction. Compared with other studies, Huang (39) used a shorter SFI single-cycle (7 d), the effect of Yiqi Fuzheng Jianpi was not obvious, and the dose of cisplatin was high (100 mg/m^2^). The study by Wang (48), the only study using the NC protocol, was classified as high-risk as the biological sex and number of pathological types did not match the total number, indicating that there may be counting errors. The conclusion after excluding these studies was consistent with the original conclusion (RR = 0.52, 95% CI = 0.44–0.62, P < 0.00001, I^2 = ^0%).

### 4.6 Publication bias analysis

**Figure 9:**
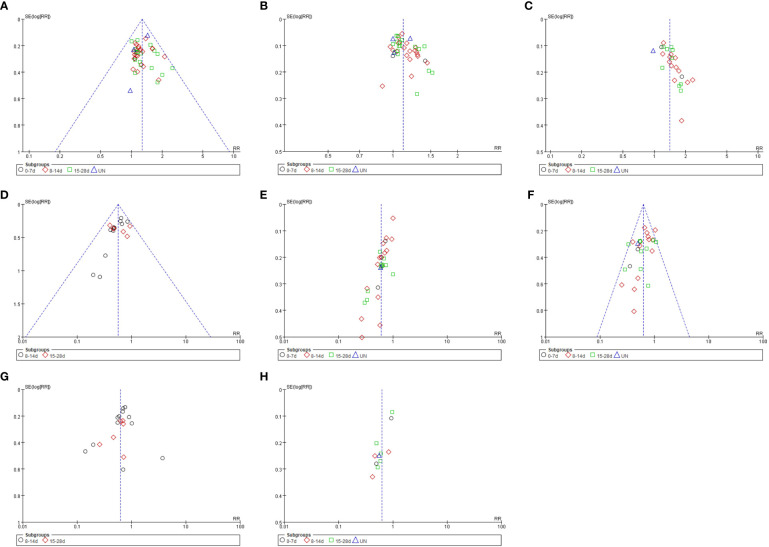
Funnel plot of analysis results. **(A)** ORR; **(B)** DCR; **(C)** KPS; **(D)** Hemoglobinia; **(E)** Leukopenia; **(F)** Thrombocytopenia; **(G)** Nausea and Vomiting; **(H)** Gastrointestinal Reaction.

More than 10 studies were included that documented the ORR, DCR, and KPS improvement rate, incidence of hemoglobin reduction, leukopenia, thrombocytopenia, nausea, and vomiting, and simple gastrointestinal reaction of SFI combined with PBC in the treatment of advanced NSCLC. A funnel plot was plotted based on the data of these studies, with the RR value as the abscissa and the logarithmic standard error SE (logRR) of RR value as the ordinate (**Figure 9**). The funnel plot showed asymmetry and skewed distribution, suggesting that there may be potential publication bias or low methodological quality, which may be related to the difficulty of publishing negative results, small sample size of some studies, different chemotherapy regimens of the control group, different intervention doses, and different courses of treatment.

### 4.7 Sensitivity analysis

Eight high-risk studies (29, 35, 43, 48, 55, 60, 61, 65) were excluded from sensitivity analysis, and the ORR results did not change significantly. The difference in the effective rate of SFI combined with PBC for the treatment of advanced NSCLC was statistically significant (RR = 1.26, 95% CI = 1.17–1.37, P < 0.00001). After the exclusion of 16 studies (31, 40, 48, 52–57, 59–61, 64, 67, 68, 70) published before 2010, the ORR results did not significantly change (RR = 1.29, 95% CI = 1.18–1.41, P < 0.00001), indicating that the meta-analysis results were stable and the conclusions were reliable.

## 5 Discussion

### 5.1 Efficacy analysis

#### 5.1.1 Overall analysis

This paper systematically evaluated the efficacy and safety of SFI combined with PBC for the treatment of advanced NSCLC. The results showed that SFI combined with PBC had advantages in improving ORR, DCR, and quality of life and could improve clinical symptoms. At the same time, SFI adjuvant chemotherapy could reduce bone marrow suppression such as hemoglobin reduction, leukopenia, and thrombocytopenia, as well as gastrointestinal adverse reactions such as nausea, vomiting, and diarrhea, which helps to improve patient compliance and treatment confidence. In general, SFI synergistic chemotherapy reduced toxicity and increased efficiency, which was consistent with previous studies. Sensitivity analysis suggested that the results of the meta-analysis were stable. Modern pharmacological studies have shown that the Astragalus polysaccharide in *A. membranaceus* has immune regulation effects and can activate non-specific immunity. It may affect the tumor inflammatory microenvironment through the TLR4/MyD88/NF-κB signaling pathway and regulation of extracellular matrix (71), affecting tumor cell apoptosis and tissue metabolism. Ginsenosides in *C. pilosula* have been shown to improve macrophage function, reduce fatigue, inhibit tumor angiogenesis, and regulate nerves. By inhibiting the expression of the Keap1-Nrf2/ARE signaling pathway, STAT3/c-myc pathway, and key enzymes of glycolysis, ginsenosides can significantly inhibit the proliferation of NSCLC cells, promote apoptosis (72, 73), effectively reduce the level of VEGF in serum, and reverse drug resistance (74). The combination of *A. membranaceus* and *C. pilosula* plays a role in reducing toxicity and increasing efficiency and comprehensive regulation in tumor treatment, which embodies the idea of “strengthening the body resistance and eliminating pathogenic factors” in traditional Chinese medicine. SFI has been shown to reduce the expression of VEGF and SIL-2R, promote the expression of IL-2 and IFN-γ, improve the cellular immune function of patients (increase of NK, CD3^+^, and CD4^+^ cells), reduce the levels of CEA, CA125 and CA19-9, and exert anti-tumor effects, prolonging survival (75, 76). Studies have shown that Astragalus membranaceus can enhance musclar hypertrophy by increasing PI3K/Akt/mTOR signaling phosphorylation, increase the diameter and thickness of myotubes by 1.16 times,and maintain muscle structure and force production (77). Therefore, SFI can be used for clinical adjuvant chemotherapy for the treatment of NSCLC, especially for patients with lung and spleen qi deficiency.

#### 5.1.2 Subgroup analysis

This study conducted a stratified analysis based on the number of days of single-cycle SFI medication, especially in improving the quality of life of patients with significant time correlation. According to the number of days of single-cycle SFI medication, we divided treatments into three subgroups: 0–7, 8–14, and 15–28 d. The results showed that 0–7 d subgroup had no significant improvement in ORR, DCR, KPS, and simple gastrointestinal reaction, but the improvement of thrombocytopenia was better than that of single-cycle long-term medication. Treatment for 8–14 d was advantageous in improving KPS, hemoglobin reduction incidences, and gastrointestinal adverse reactions. Treatment for 15–28 d had the most significant improvement in ORR, leukopenia incidences, and nausea and vomiting incidences. Therefore, prolonging the single-cycle SFI medication time could improve multiple outcome indicators. Based on these findings, we recommend that the single-cycle SFI medication time should be 15–28 d, which was the most beneficial length for tumor adjuvant therapy. The second recommendation is 8–14 d, which was most beneficial for improving the quality of life of patients and reducing adverse reactions. SFI combined with PBC could significantly reduce the incidence of bone marrow suppression (including the incidence of hemoglobin reduction, leukopenia, and thrombocytopenia), regardless of the length of single-cycle medication. This may be due to the direct protection of hematopoietic stem cells by astragalus polysaccharides and the promotion of hematopoietic stem cell development by regulating FOS gene expression (78). Animal experiments have shown that ginsenosides promote hematopoietic cell proliferation and differentiation by regulating GATA transcription factors in mouse bone marrow cells (79), which is consistent with the conclusions of this and previous studies (80). The results suggest that SFI has good clinical application value in adjuvant PBC for improving bone marrow suppression (**Table 5**).

**Table 5:** Result summary table.

Group	ORR	DCR	KPS	Hemoglobinia	Leukopenia	Thrombo-cytopenia	Myelosup-pression	Nausea and vomiting	Diarrhea	Gastrointestinal Reaction
Subgroups divided according to the duration of single-cycle SFI										
0-7d	N	N	N	U	Y	Y	Unclassified	U	Unclassified	N
8-14d	Y	Y	Y	Y	Y	Y		Y		Y
15-28d	Y	Y	Y	Y	Y	Y		Y		Y
UN	Y	N	U	U	Y	U		U		U
Total	Y	Y	Y	Y	Y	Y	Y	Y	Y	Y
Heterogeneity	P=0.98 I²=0%	P=0.35 I²=6%	P=0.04 I²=35%	P=0.78 I²=0%	P<0.00001 I²=77%	P=0.12 I²=25%	P=0.53 I²=0%	P=0.0002 I²=63%	P=0.11 I²=47%	P<0.00001 I²=78%
Test for subgroup differences	P=0.73 I²=0%	P=0.93 I²=0%	P=0.02 I²=70.4%	P=0.87 I²=0%	P=0.81 I²=0%	P=0.36 I²=6.6%	U	P=0.58 I²=0%	U	P=0.93 I²=0%
Subgroups divided according to the chemotherapy plan										
SFI+GP/GP	Y	Y	Y	Y	Y	Y	Unclassified	N	Unclassified	Y
SFI+NP/NP	Y	Y	Y	Y	Y	Y		N		N
SFI+TP/TP	Y	Y	Y	Y	Y	Y		Y		Y
SFI+DP/DP	N	N	N	Y	Y	Y		N		N
SFI+NC/NC	N	N	Y	N	Y	Y		Y		N
SFI+GC/GC	Y	U	Y	U	U	U		U		U
SFI+AP/AP	N	N	U	U	N	N		Y		U
SFI+TC/TC	N	N	Y	U	U	U		N		U
Total	Y	Y	Y	Y	Y	Y	Y	Y	Y	Y
Test for subgroup differences	P=0.81 I²=0%	P=0.14 I²=38.4%	P=0.16 I²=34.8%	P=0.88 I²=0%	P=0.47 I²=0%	P=0.33 I²=13.0%	U	P=0.36 I²=9.5%	U	P=0.003 I²=75.4%

Y, statistically significant difference between the test group and the control group; N, no statistically significant difference between the test group and the control group; U, no relevant data in the included literature, or only 1 piece of literature, which could not be analyzed. GP, gemcitabine + cisplatin; NP, vinorelbine + cisplatin; TP, paclitaxel/albumin paclitaxel/paclitaxel liposome + cisplatin; DP, docetaxel + cisplatin; NC, vinorelbine + carboplatin; GC, gemcitabine + carboplatin; AP, pemetrexed + cisplatin; TC, paclitaxel/albumin paclitaxel/paclitaxel liposome + carboplatin. Objective remission rate ORR=(CR+PR)/total cases×100%; Disease control rate DCR=(CR+PR+SD)/total cases×100%; KPS improvement rate=(number of improved cases + number of stable cases)/total cases.

According to the subgroup analysis of the specific chemotherapy type, SFI combined with GP, NP, TP, and GC significantly improved the curative effect and the quality of life of patients. SFI combined with GP, NP, TP, DP, and NC regimens could significantly reduce bone marrow suppression. For ORR, SFI combined with GP, GC, and TP groups had the most obvious advantages. For DCR, the effect was greatest in the SFI + TP group, while the combination with NC, DP, and TC was not recommended. In terms of improving the quality of life, SFI combined with TP, GC, and TC showed obvious advantages. However, GC and TC regimens were reported in only one study each, therefore further studies are required to confirm the beneficial effects. In terms of reducing myelosuppression, the SFI + TP regimen had a clear advantage, while SFI combined with NP, DP, and NC regimens were not recommended. In general, SFI was the most effective for patients treated with the TP regimen, with obvious significance for reducing bone marrow suppression and improving gastrointestinal reactions. However, the outcome indicators of the literature included in this study are quite different, and some have no relevant data, so it is impossible to make a comprehensive comparative analysis.

**Figure 10:**
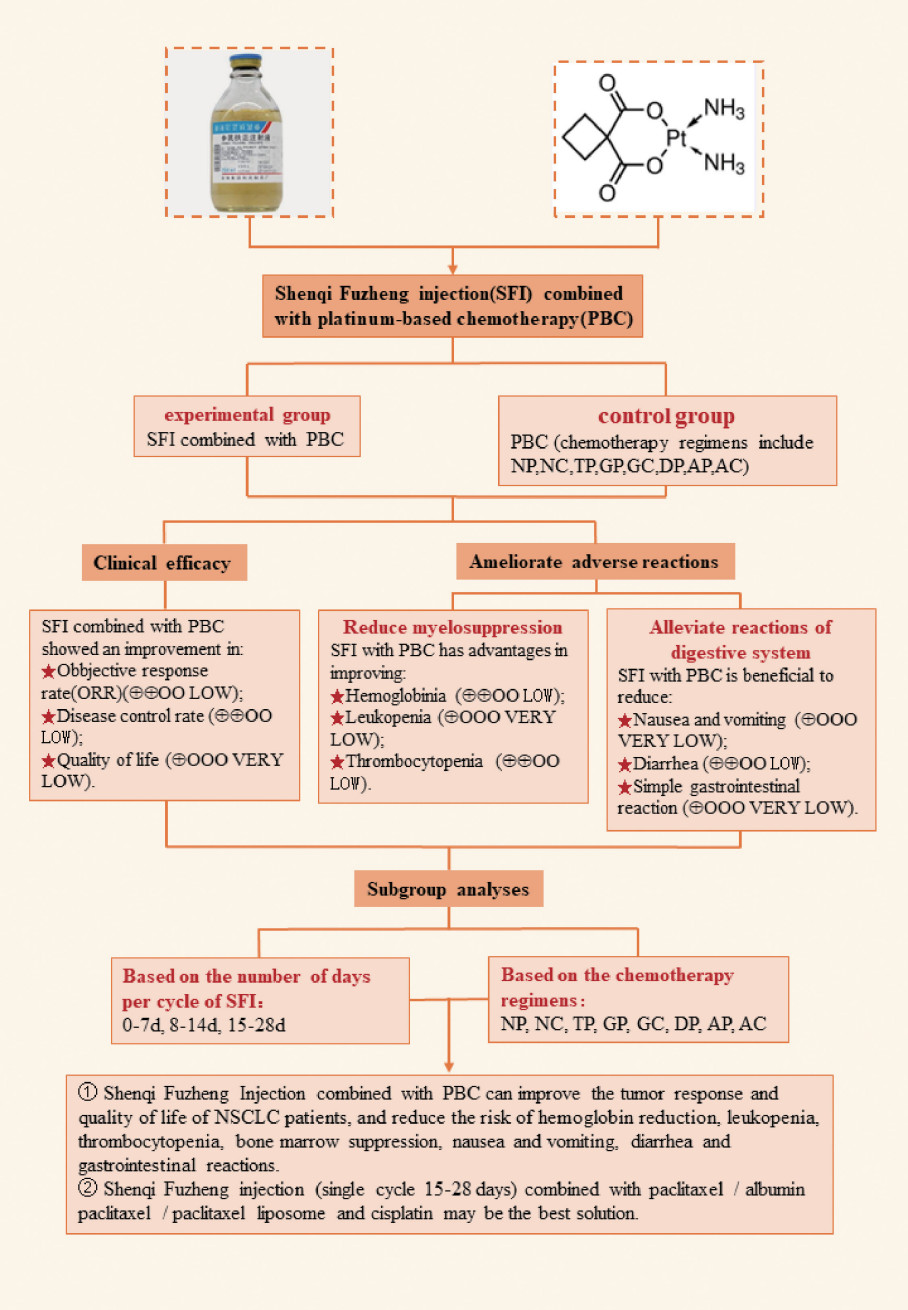
SFI combined with PBC for non-small cell lung cancer. NP, vinorelbine + cisplatin; NC, vinorelbine + carboplatin; TP, paclitaxel/albumin paclitaxel/paclitaxel liposome + cisplatin; TC, paclitaxel/albumin paclitaxel/paclitaxel liposome + carboplatin; GP, gemcitabine + cisplatin; GC, gemcitabine + carboplatin; DP, docetaxel + cisplatin; DC, docetaxel + carboplatin; AP, pemetrexed + cisplatin; AC, pemetrexed + carboplatin.

In summary, combining results for ORR, DCR, improvement of quality of life, and adverse reactions, we recommend a single-cycle of SFI medication for 15–28 d combined with the TP regimen to achieve the most beneficial outcomes (**Figure 10**).

The heterogeneity test analysis showed that, except for the two studies (54, 59) in the leukocyte group, heterogeneity was not obvious in the short-term efficacy, quality of life evaluation, and bone marrow suppression. However, the heterogeneity of digestive tract reaction was obvious. This may be because the dosage of chemotherapy was quite different, digestive tract reactions have individual differences, and it is susceptible to non-chemotherapy factors. However, SFI adjuvant chemotherapy still had a clear remission effect on gastrointestinal adverse reactions.

### 5.2 Limitations of this study

There are a number of limitations to the meta-analysis based on the chemotherapy regimen. (1) The vast majority of source reports use more male patients than women, and the ratio of male to female will affect the results. However, most of the current experimental designs do not take into account biological sex differences, so this article may have certain limitations on biological sex factors. In the subsequent design of RCTs, male and female outcome indicators should be described separately to further explore the biological sex differences in SFI efficacy. (2) Along with stage and metastases, weight loss is closely tied to mortality in patients with NSCLC. But the studies did not report post-treatment weight, and most of the studies only had baseline data on weight. Therefore the meta-analysis could not summarize the weight change before and after treatment.The inability to report whether SFI combined with chemotherapy has any effect on the prevention of weight loss is one of the limitations of this paper.(3)The literature included in the study was limited to single-center studies, and no reference was made to the basis of sample size estimation. The minimum sample size was 36, and the median sample size was 80. Often, the number of studies included in the subgroup analysis was small and there was a certain degree of heterogeneity among the studies. This possibly resulted in bias in the study results and reduced test efficacy. (4) Random allocation was mentioned in the included literature, but 23 studies did not describe the specific random sequence generation method. Except for one study using the envelope method, there was no mention of whether allocation concealment was implemented. Therefore, there were some limitations in methodology, which meant the existence of selective bias could not be ruled out and may have affected the accuracy of the results. (5) Implementing blind methodology with randomization in clinical trials of chemotherapy and traditional Chinese medicine injection is difficult, and this method was not mentioned in the literature. This means the results may be subjectively affected by patients, implementers, and outcome measurers, causing implementation and measurement bias. (6) Literature bias analysis showed the inverted funnel plots of KPS, leukopenia incidence, and thrombocytopenia incidence were asymmetrically distributed, suggesting that there may be publication bias; the efficacy of SFI combined chemotherapy needs further study and verification. (7) No adverse reactions caused by SFI were noted in the included literature; observations of SFI safety in clinical application needs to be improved. In summary, the methodology and research quality of the literature included in this study were generally low. The above limitations may reduce the stability and reliability of the results, and affect the recommendation level and evidential support of the system evaluation.

### 5.3 Future research possibilities

There are a number of areas that would benefit from further research. (1) In subsequent studies we can design high-quality RCTs, using weight change and/or cachexia in patients with NSCLC as observational indicators to explore the preventive and curative effects of SFI and to observe whether patients with weight loss respond differently to treatment than controls. (2) Currently there are more RCTs of SFI combined with platinum-based chemotherapy in China, while there are fewer RCTs of combined radiotherapy. At the same time, in China, the treatment of NSCLC with SFI is mostly combined in the chemotherapy stage, while the radiotherapy stage is mostly treated with compound matrine injection. Therefore, the systematic evaluation of SFI combined with radiotherapy for NSCLC has certain research value. (3) To conclusively verify the results of the existing clinical RCTs, studies need to further expand the sample size, improve the quality of clinical trials, conduct a standardized and comprehensive design, or carry out high-quality multicenter randomized double-blind trials. (4) Strict randomization and allocation concealment methods should be used in clinical research, and RCTs should incorporate explicit reporting of randomization implementation methods when conducting systematic evaluations. When the double-blindness of subjects and researchers cannot be achieved, blinding of evaluators can be implemented to further improve the objectivity of the results. (5) The dosage, frequency, and cycle of SFI and chemotherapy drugs should be standardized to reduce heterogeneity. This will facilitate accurate comparisons to understand the role of SFI. (6) Adverse reactions should be fully reported and the clinical safety of traditional Chinese medicine injections requires greater attention to provide evidence for rational drug use. (7) RCT reports should be carried out according to the Consort standard as far as possible (81), and the outcome indicators should be reported truthfully to obtain more reliable research results. (8) Long-term follow-up studies should be carried out following clinical trials to report comprehensive and meaningful outcome and endpoint indicators. Further research should be carried out on whether combined treatment can improve the long-term survival rate, efficacy, and the quality of life of patients, for scientifically guided clinical decision-making. (9) The results of this study showed that, compared with other chemotherapy regimens, the efficacy of SFI combined with TP regimen was more obvious in all aspects. Investigations into whether there is a specific mechanism that increases the synergy of SFI with TP would be valuable.

### 5.4 Conclusion

In summary, the incidence and mortality of lung cancer are high in the world. Platinum-based doublet chemotherapy is the first-line standard treatment, but the efficacy of chemotherapy is limited and the side effects are large, which affects the quality of life of patients.The treatment of advanced NSCLC was improved with by using SFI combined with PBC compared with PBC alone. SFI combined with PBC could significantly improve the clinical efficiency and quality of life, while reducing adverse reactions and improving thee safety. Use of SFI with PBC has high research value and wide application prospects. A total of 44 RCTs were included in this study, with a total of 3460 patients. Compared with the existing research, the latest research is supplemented, and more comprehensive search and inclusion studies are included.So the results were more objective. In this paper, subgroup analysis was carried out according to the number of days of single-cycle SFI medication and the combined chemotherapy regimen, and the optimal number of days of single-cycle SFI medication and the optimal chemotherapy regimen combined with SFI were obtained. This is not perfect in previous studies, but also the most significant improvement in this paper.However, this study has limitations such as low quality of the included literature, small sample size, and insufficient standardization and rigorous experimental design. In order to further verify SFI efficacy and adverse reactions, multicenter, large sample, scientific, and standardized RCTs and basic research are needed to provide higher quality medical evidence.

## Data availability statement

The original contributions presented in the study are included in the article/**Supplementary Material**. Further inquiries can be directed to the corresponding author.

## Author contributions

Conception and design: WH, ZL. Provision of study material or patients: All authors. Collection and assembly of data: All authors. Data analysis and interpretation: CQ, SH, DW, KC, ZW, XW, XM. Manuscript writing: CQ. All authors contributed to the article and approved the submitted version.
