# Supplementary material for: Effectiveness and safety of Shenqi Fuzheng injection combined with platinum-based chemotherapy for treatment of advanced non-small cell lung cancer: a systematic review and meta-analysis
Source: Front Oncol. 2023 Aug 24;13:1198768. doi: 10.3389/fonc.2023.1198768 (PMC10507621; doi:10.3389/fonc.2023.1198768)
Supplement: Supplementary file 1 [file Presentation_1.pdf]

Supplementary Material

1 Search process and search strategy

2.1 Search strategy of CNKI

(SU= Chinese medicine injection OR SU= Chinese medicine injection OR SU= Shenqi Fuzheng injection OR SU= Shenqi Fuzheng injection OR SU= Shenqi Fuzheng injection OR SU= Shenqi Fuzheng injection OR SU= non-small cell lung cancer OR SU= lung cancer OR SU= lung tumor OR SU= lung tumor OR SU=NSCLC) NOT (TI= mouse OR TI= rabbit)

2.2 Search strategy of VIP

(M= Chinese medicine injection OR M= Chinese medicine injection OR M= Shenqi Fuzheng injection OR M= Shenqi Fuzheng injection OR M= Shenqi Fuzheng injection OR M= Shenqi Fuzheng injection OR M= Shenqi Fuzheng injection OR M= non-small cell lung cancer OR M= lung cancer OR M= lung tumor or M= lung tumor or M=NSCLC) NOT (T= mouse OR T= rabbit)

2.3 Search strategy of Wanfang data

Subject :(" TCM Injection "or" TCM Injection "or" Shenqi Fuzheng Injection "or" Shenqi Fuzheng Injection "or" Shenqi Fuzheng Injection "or" Shenqi Fuzheng Liquid ") and subject :(" non-small cell lung cancer "or" lung cancer "or" lung tumor "or" NSCLC ") not (Title: "Rabbit" or "Rat")

2.4 Search strategy of SinoMed

(" Cancer, Non-small cell lung "[unweighted: extended] OR "Lung tumor "[unweighted: extended] OR" non-small cell lung cancer "[common field: intelligent] OR "non-small cell lung cancer "[common field: intelligent] OR "NSCLC"[common field: intelligent] OR" lung cancer "[common field: intelligent] OR "Lung cancer "[common field: intelligent] OR" lung tumor "[common field: intelligent] OR "lung tumor "[common field: intelligent]) AND (" Chinese medicine injection "[common field: intelligent] OR" Chinese medicine injection "[common field: intelligent] OR "Shenqi Fuzheng

injection "[common field: intelligent] OR "Shenqi Fuzheng Injection "[common field: intelligent] OR" Shenqi Fuzheng injection "[common field: intelligent] OR "Shenqi Fuzheng liquid "[common field: intelligent])

2.5 Search strategy of Pubmed

#1 Carcinoma, Non-Small-Cell Lung [MeSH] OR Carcinoma, Non Small Cell Lung[tiab] OR Carcinomas, Non-Small-Cell Lung[tiab] OR Lung Carcinoma, Non-Small-Cell[tiab] OR Lung Carcinomas, Non-Small-Cell[tiab] OR Non-Small-Cell Lung Carcinomas[tiab] OR Non-Small-Cell Lung Carcinoma[tiab] OR Non Small Cell Lung Carcin[i]oma[tiab] OR Carcinoma, Non-Small Cell Lung[tiab] OR Non-Small Cell Lung Carcinoma[tiab] OR Non-Small Cell Lung Cancer[tiab] OR Nonsmall Cell Lung Cancer[tiab] OR NSCLC[tiab]

#2 (tumor*[tiab] OR carcinoma*[tiab] OR neoplasm*[tiab] OR cancer*[tiab]) AND (lung[tiab])

#3 #1 OR #2

#4 Chinese herbal injection*[tiab] OR Chinese medicine injection[tiab] OR injection of TCM[tiab] OR Shenqi Fuzheng[tiab] OR Shenqi Fuzheng injection[tiab] OR Shenqifuzheng injection[tiab] OR Ginseng-Qi Fuzheng injection[tiab] OR SFI[tiab]

#5#3 AND #4

2.6 Search strategy of Cochrane library

#1 MeSH descriptor: [Carcinoma, Non-Small-Cell Lung] explode all trees

#2 ((tumor* OR carcinoma* OR neoplasm* OR cancer*) AND (lung)):ti,ab,kw

#3 #1 OR #2

#4 Chinese herbal injection*:ti,ab,kw or Chinese medicine injection:ti,ab,kw or injection of TCM:ti,ab,kw or Shenqi Fuzheng:ti,ab,kw or Shenqifuzheng:ti,ab,kw or Ginseng-Qi Fuzheng:ti,ab,kw or SFI:ti,ab,kw

#5 #3 AND #4

2.7 Search strategy of Embase

#1 'non small cell lung cancer'/exp OR 'non small cell lung cancer'

#2 (tumor*:ab,kw,ti OR carcinoma*:ab,kw,ti OR neoplasm*:ab,kw,ti OR cancer*:ab,kw,ti) AND lung:ab,kw,ti

#3 #1 OR #2

#4 'chinese herbal injection*':ab,kw,ti OR 'chinese medicine injection':ab,kw,ti OR 'injection of tcm':ab,kw,ti OR 'Shenqi Fuzheng injection':ab,kw,ti OR ' Shenqifuzheng ':ab,kw,ti OR ' Ginseng-Qi Fuzheng injection':ab,kw,ti OR ' SFI':ab,kw,ti

#5 #3 AND #4

3 supplementary figures

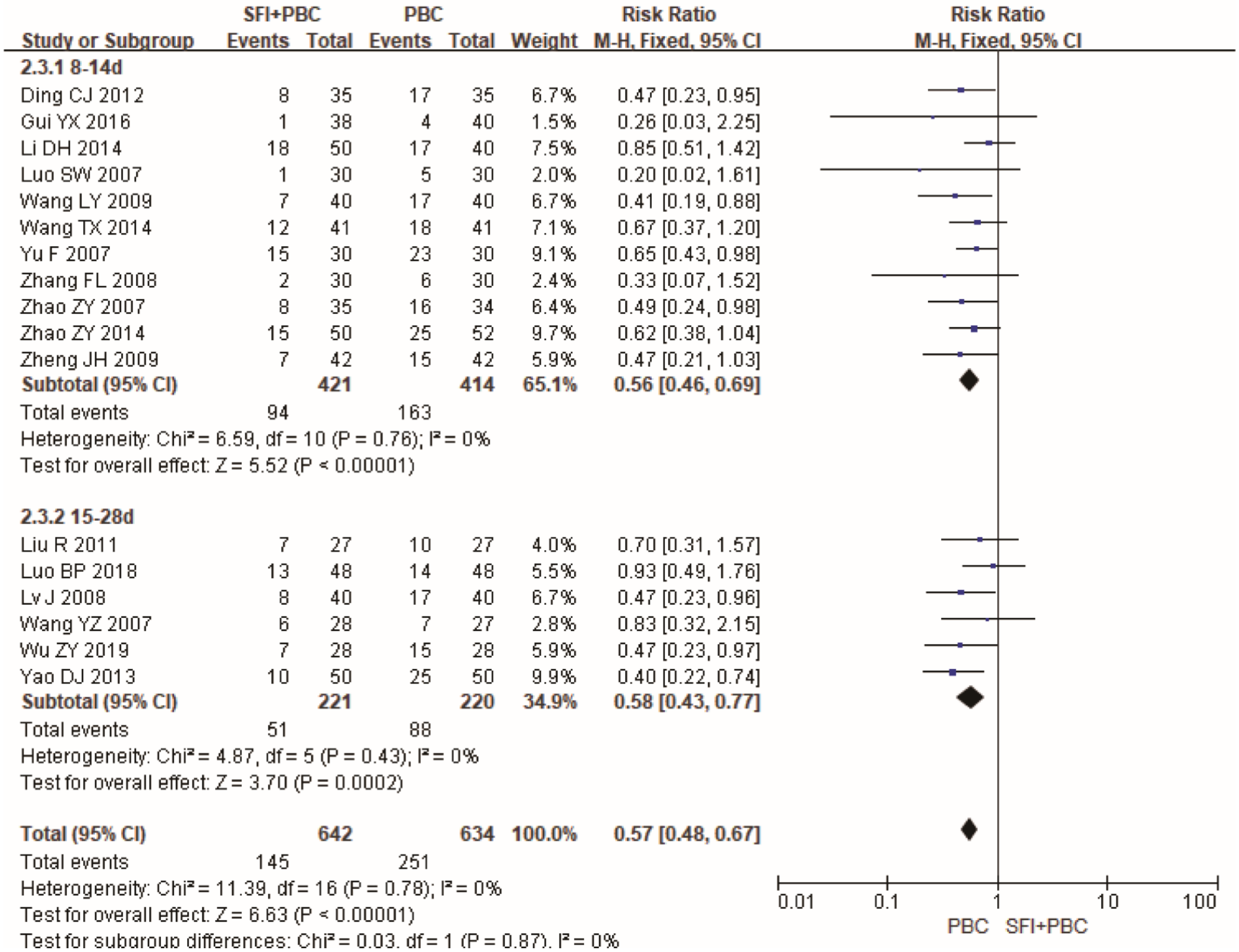

Supplementary Figure 1 Forest plot of the incidence of hemoglobin reduction stratified by days of single-cycle SFI dosing

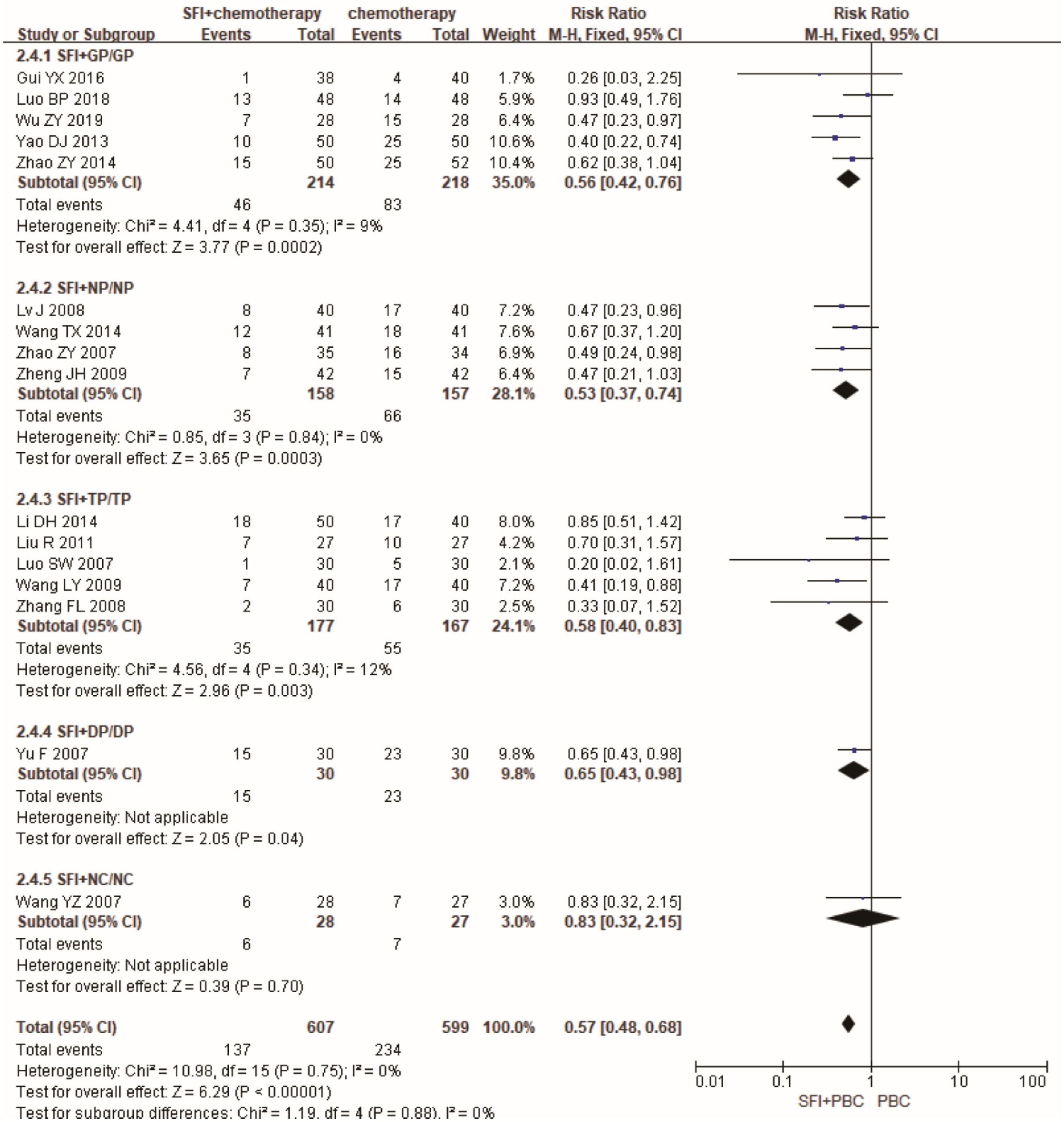

Supplementary Figure 2 Forest plot of hemoglobinopenia incidence stratified by chemotherapy regimen

Note: GP,gemcitabine + cisplatin; NP,vinorelbine + cisplatin; TP,paclitaxel / albumin paclitaxel / paclitaxel liposome + cisplatin; DP,docetaxel + cisplatin ; NC,vinorelbine + carboplatin.

Study or Subgroup	SFI+PBC Events	SFI+PBC Total	PBC Events	PBC Total	Weight	Risk Ratio M-H, Random, 95% CI
**2.5.1 0-7d**						
Huang AX 2014	24	38	33	38	4.3%	0.73 [0.55, 0.96]
Ma CG 2013	9	28	17	28	2.7%	0.53 [0.29, 0.98]
**Subtotal (95% CI)**		**66**		**66**	**7.0%**	**0.69 [0.54, 0.89]**
Total events	33		50			
Heterogeneity: Tau² = 0.00; Chi² = 0.98, df = 1 (P = 0.32); I² = 0%						
Test for overall effect: Z = 2.91 (P = 0.004)						
**2.5.2 8-14d**						
Ding CJ 2012	14	35	27	35	3.5%	0.52 [0.33, 0.81]
Ding PQ 2016	22	60	36	60	3.7%	0.61 [0.41, 0.90]
Gui YX 2016	6	38	11	40	1.8%	0.57 [0.24, 1.40]
Jia YL 2012	40	72	52	71	4.4%	0.76 [0.59, 0.97]
Li DH 2014	20	50	28	40	3.7%	0.57 [0.38, 0.85]
Luo SW 2007	4	30	15	30	1.6%	0.27 [0.10, 0.71]
Ren JS 2015	9	42	17	42	2.4%	0.53 [0.27, 1.05]
Wang K 2007	18	18	18	18	4.9%	1.00 [0.90, 1.11]
Wang LY 2009	18	40	29	40	3.7%	0.62 [0.42, 0.92]
Wang TX 2014	16	41	25	41	3.4%	0.64 [0.41, 1.01]
Yu F 2007	17	30	24	30	3.9%	0.71 [0.49, 1.02]
Yu QZ 2007	23	30	26	32	4.4%	0.94 [0.73, 1.22]
Zhang FL 2008	5	30	19	30	1.9%	0.26 [0.11, 0.61]
Zhao ZY 2007	21	35	31	34	4.2%	0.66 [0.49, 0.88]
Zhao ZY 2014	25	50	34	52	4.0%	0.76 [0.54, 1.07]
Zheng JH 2009	9	42	27	42	2.7%	0.33 [0.18, 0.62]
**Subtotal (95% CI)**		**643**		**637**	**54.3%**	**0.62 [0.49, 0.78]**
Total events	267		419			
Heterogeneity: Tau² = 0.16; Chi² = 100.05, df = 15 (P < 0.00001); I² = 85%						
Test for overall effect: Z = 4.13 (P < 0.0001)						
**2.5.3 15-28d**						
Bao Z 2019	9	47	26	47	2.6%	0.35 [0.18, 0.66]
He WX 2021	17	48	27	48	3.4%	0.63 [0.40, 0.99]
Li Y 2007	16	44	26	43	3.4%	0.60 [0.38, 0.95]
Liu R 2011	13	27	20	27	3.4%	0.65 [0.41, 1.02]
Luo BP 2018	18	48	18	48	3.1%	1.00 [0.60, 1.68]
Luo SZ 2006	6	25	20	25	2.3%	0.30 [0.15, 0.62]
Lv J 2008	16	40	26	40	3.5%	0.62 [0.40, 0.96]
Wang HL 2021	8	53	24	53	2.4%	0.33 [0.16, 0.67]
Wu ZY 2019	14	28	19	28	3.5%	0.74 [0.47, 1.16]
Yao DJ 2013	22	50	38	50	3.9%	0.58 [0.41, 0.82]
Zhao Q 2019	21	52	31	52	3.7%	0.68 [0.45, 1.01]
**Subtotal (95% CI)**		**462**		**461**	**35.3%**	**0.59 [0.50, 0.71]**
Total events	160		275			
Heterogeneity: Tau² = 0.03; Chi² = 14.44, df = 10 (P = 0.15); I² = 31%						
Test for overall effect: Z = 5.82 (P < 0.00001)						
**2.5.4 UN**						
Jia J 2020	15	40	25	40	3.4%	0.60 [0.38, 0.96]
**Subtotal (95% CI)**		**40**		**40**	**3.4%**	**0.60 [0.38, 0.96]**
Total events	15		25			
Heterogeneity: Not applicable						
Test for overall effect: Z = 2.15 (P = 0.03)						
**Total (95% CI)**		**1211**		**1204**	**100.0%**	**0.61 [0.53, 0.71]**
Total events	475		769			
Heterogeneity: Tau² = 0.12; Chi² = 126.68, df = 29 (P < 0.00001); I² = 77%						
Test for overall effect: Z = 6.49 (P < 0.00001)						
Test for subgroup differences: Chi² = 0.95, df = 3 (P = 0.81), I² = 0%						

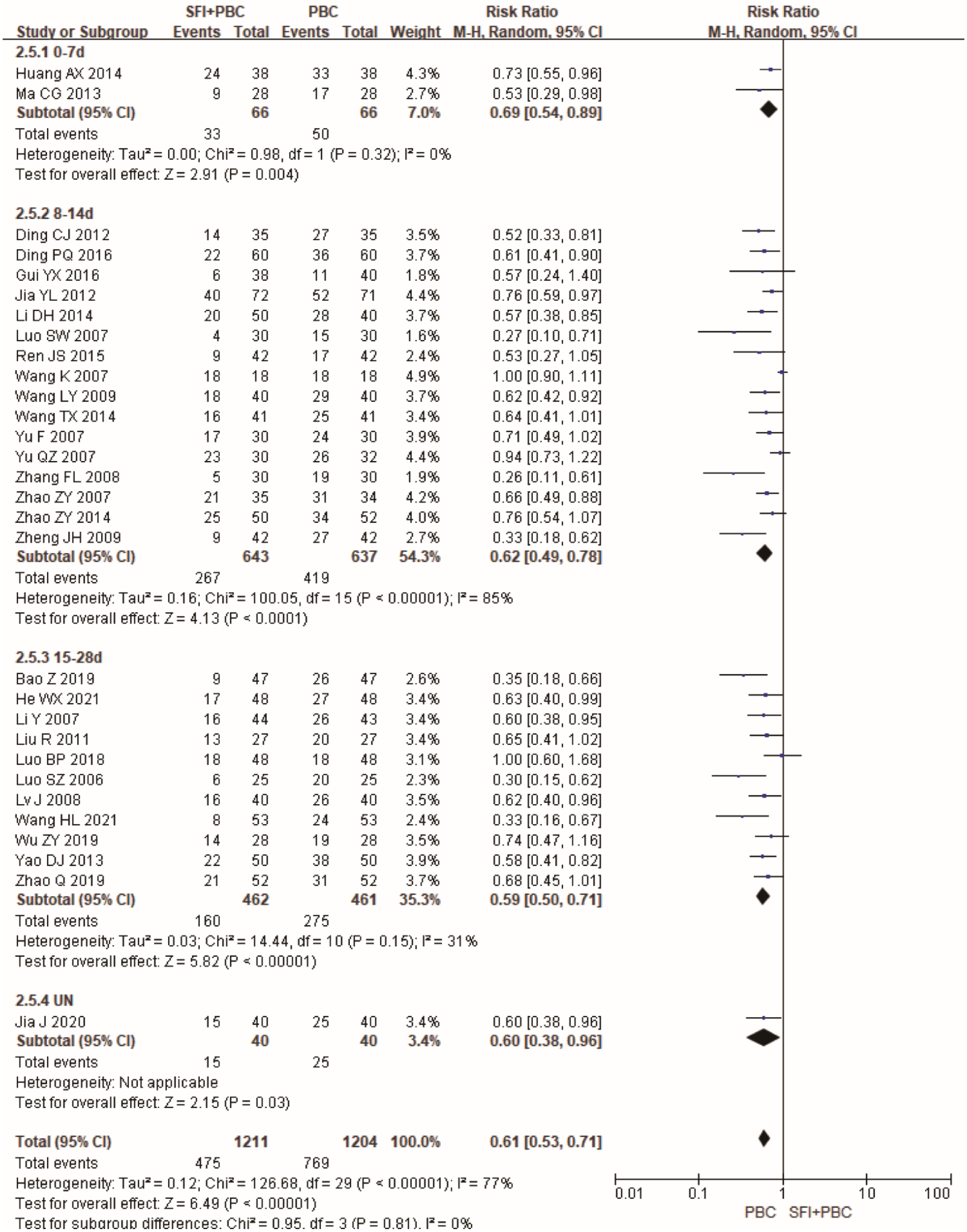

Supplementary Figure 3 Forest plot of incidence of leukopenia stratified by days of single-cycle SFI dosing

Study or Subgroup	SFI+chemotherapy Events	SFI+chemotherapy Total	chemotherapy Events	chemotherapy Total	Weight	Risk Ratio M-H, Random, 95% CI
**2.6.1 SFI+GP/GP**						
Bao Z 2019	9	47	26	47	2.1%	0.35 [0.18, 0.66]
Gui YX 2016	6	38	11	40	1.2%	0.57 [0.24, 1.40]
He WX 2021	17	48	27	48	3.5%	0.63 [0.40, 0.99]
Huang AX 2014	24	38	33	38	6.6%	0.73 [0.55, 0.96]
Jia J 2020	15	40	25	40	3.4%	0.60 [0.38, 0.96]
Luo BP 2018	18	48	18	48	2.9%	1.00 [0.60, 1.68]
Wang HL 2021	8	53	24	53	1.8%	0.33 [0.16, 0.67]
Wu ZY 2019	14	28	19	28	3.6%	0.74 [0.47, 1.16]
Yao DJ 2013	22	50	38	50	5.0%	0.58 [0.41, 0.82]
Zhao ZY 2014	25	50	34	52	5.2%	0.76 [0.54, 1.07]
**Subtotal (95% CI)**		**440**		**444**	**35.3%**	**0.64 [0.54, 0.76]**
Total events	158		255			
Heterogeneity: Tau² = 0.02; Chi² = 12.83, df = 9 (P = 0.17); I² = 30%						
Test for overall effect: Z = 5.05 (P < 0.00001)						
**2.6.2 SFI+NP/NP**						
Ding PQ 2016	22	60	36	60	4.4%	0.61 [0.41, 0.90]
Li Y 2007	16	44	26	43	3.5%	0.60 [0.38, 0.95]
Lv J 2008	16	40	26	40	3.7%	0.62 [0.40, 0.96]
Wang K 2007	18	18	18	18		Not estimable
Wang TX 2014	16	41	25	41	3.6%	0.64 [0.41, 1.01]
Yu QZ 2007	23	30	26	32	6.9%	0.94 [0.73, 1.22]
Zhao ZY 2007	21	35	31	34	6.2%	0.66 [0.49, 0.88]
Zheng JH 2009	9	42	27	42		Not estimable
**Subtotal (95% CI)**		**250**		**250**	**28.2%**	**0.70 [0.58, 0.84]**
Total events	114		170			
Heterogeneity: Tau² = 0.02; Chi² = 7.29, df = 5 (P = 0.20); I² = 31%						
Test for overall effect: Z = 3.92 (P < 0.0001)						
**2.6.3 SFI+TP/TP**						
Li DH 2014	20	50	28	40	4.3%	0.57 [0.38, 0.85]
Liu R 2011	13	27	20	27	3.6%	0.65 [0.41, 1.02]
Luo SW 2007	4	30	15	30	1.0%	0.27 [0.10, 0.71]
Luo SZ 2006	6	25	20	25	1.7%	0.30 [0.15, 0.62]
Wang LY 2009	18	40	29	40	4.3%	0.62 [0.42, 0.92]
Zhang FL 2008	5	30	19	30	1.3%	0.26 [0.11, 0.61]
Zhao Q 2019	21	52	31	52	4.3%	0.68 [0.45, 1.01]
**Subtotal (95% CI)**		**254**		**244**	**20.4%**	**0.52 [0.40, 0.68]**
Total events	87		162			
Heterogeneity: Tau² = 0.05; Chi² = 10.32, df = 6 (P = 0.11); I² = 42%						
Test for overall effect: Z = 4.90 (P < 0.00001)						
**2.6.4 SFI+DP/DP**						
Ma CG 2013	9	28	17	28	2.2%	0.53 [0.29, 0.98]
Yu F 2007	17	30	24	30	4.8%	0.71 [0.49, 1.02]
**Subtotal (95% CI)**		**58**		**58**	**7.1%**	**0.66 [0.48, 0.90]**
Total events	26		41			
Heterogeneity: Tau² = 0.00; Chi² = 0.69, df = 1 (P = 0.41); I² = 0%						
Test for overall effect: Z = 2.64 (P = 0.008)						
**2.6.5 SFI+NC/NC**						
Jia YL 2012	40	72	52	71	7.1%	0.76 [0.59, 0.97]
**Subtotal (95% CI)**		**72**		**71**	**7.1%**	**0.76 [0.59, 0.97]**
Total events	40		52			
Heterogeneity: Not applicable						
Test for overall effect: Z = 2.17 (P = 0.03)						
**2.6.6 SFI+AP/AP**						
Ren JS 2015	9	42	17	42	1.9%	0.53 [0.27, 1.05]
**Subtotal (95% CI)**		**42**		**42**	**1.9%**	**0.53 [0.27, 1.05]**
Total events	9		17			
Heterogeneity: Not applicable						
Test for overall effect: Z = 1.82 (P = 0.07)						
**Total (95% CI)**		**1116**		**1109**	**100.0%**	**0.64 [0.58, 0.71]**
Total events	434		697			
Heterogeneity: Tau² = 0.02; Chi² = 38.13, df = 26 (P = 0.06); I² = 32%						
Test for overall effect: Z = 8.69 (P < 0.00001)						
Test for subgroup differences: Chi² = 5.17, df = 5 (P = 0.40), I² = 3.3%						

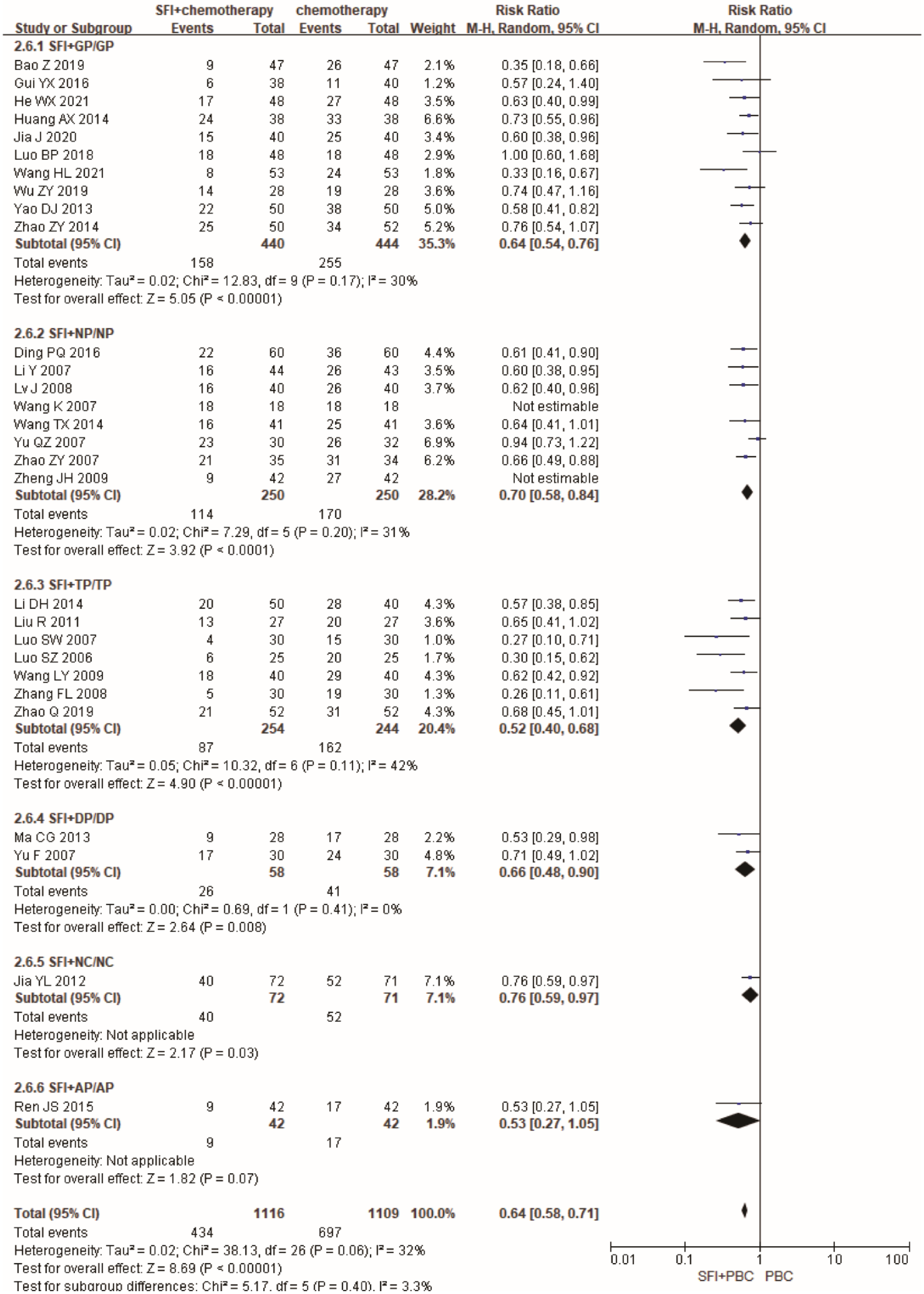

Supplementary Figure 4 Forest plot of incidence of leukopenia stratified by chemotherapy regimen

Note: GP,gemcitabine + cisplatin; NP,vinorelbine + cisplatin; TP,paclitaxel / albumin paclitaxel / paclitaxel liposome + cisplatin; DP,docetaxel + cisplatin ; NC,vinorelbine + carboplatin; AC,pemetrexed + carboplatin.

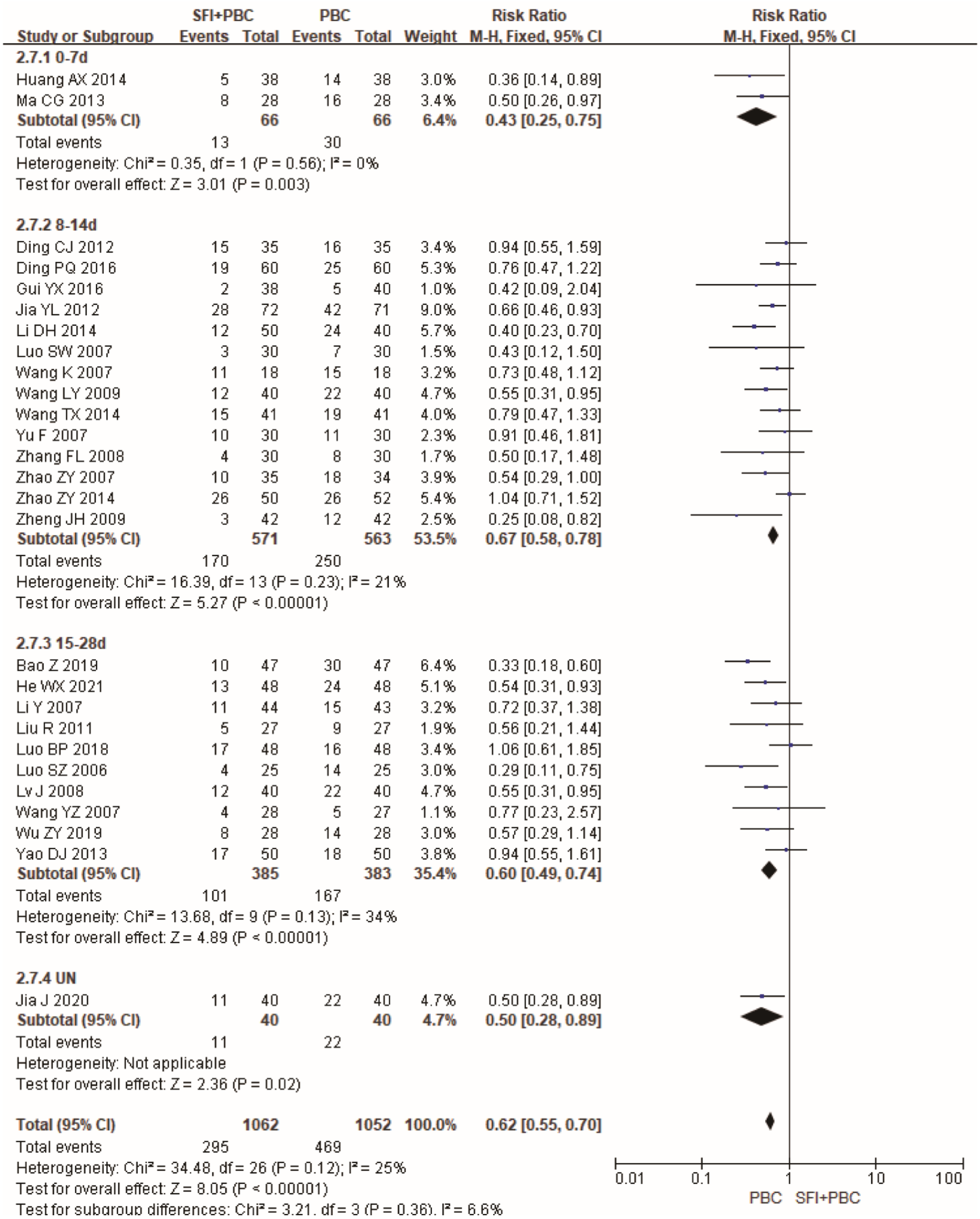

Supplementary Figure 5 Forest plot of the incidence of thrombocytopenia stratified by days of single-cycle SFI dosing

Study or Subgroup	SFI+chemotherapy Events	SFI+chemotherapy Total	chemotherapy Events	chemotherapy Total	Weight	Risk Ratio M-H, Random, 95% CI
**2.8.1 SFI+GP/GP**						
Bao Z 2019	10	47	30	47	4.2%	0.33 [0.18, 0.60]
Gui YX 2016	2	38	5	40	0.7%	0.42 [0.09, 2.04]
He WX 2021	13	48	24	48	4.8%	0.54 [0.31, 0.93]
Huang AX 2014	5	38	14	38	2.0%	0.36 [0.14, 0.89]
Jia J 2020	11	40	22	40	4.4%	0.50 [0.28, 0.89]
Luo BP 2018	17	48	16	48	4.7%	1.06 [0.61, 1.85]
Wu ZY 2019	8	28	14	28	3.3%	0.57 [0.29, 1.14]
Yao DJ 2013	17	50	18	50	4.9%	0.94 [0.55, 1.61]
Zhao ZY 2014	26	50	26	52	7.6%	1.04 [0.71, 1.52]
**Subtotal (95% CI)**		**387**		**391**	**36.7%**	**0.63 [0.46, 0.87]**
Total events	109		169			
Heterogeneity: Tau² = 0.13; Chi² = 18.96, df = 8 (P = 0.02); I² = 58%						
Test for overall effect: Z = 2.81 (P = 0.005)						
**2.8.2 SFI+NP/NP**						
Ding PQ 2016	19	60	25	60	5.7%	0.76 [0.47, 1.22]
Li Y 2007	11	44	15	43	3.6%	0.72 [0.37, 1.38]
Lv J 2008	12	40	22	40	4.7%	0.55 [0.31, 0.95]
Wang K 2007	11	18	15	18	6.7%	0.73 [0.48, 1.12]
Wang TX 2014	15	41	19	41	5.1%	0.79 [0.47, 1.33]
Zhao ZY 2007	10	35	18	34	4.0%	0.54 [0.29, 1.00]
Zheng JH 2009	3	42	12	42	1.3%	0.25 [0.08, 0.82]
**Subtotal (95% CI)**		**280**		**278**	**31.1%**	**0.67 [0.54, 0.82]**
Total events	81		126			
Heterogeneity: Tau² = 0.00; Chi² = 4.67, df = 6 (P = 0.59); I² = 0%						
Test for overall effect: Z = 3.79 (P = 0.0001)						
**2.8.3 SFI+TP/TP**						
Li DH 2014	12	50	24	40	4.6%	0.40 [0.23, 0.70]
Liu R 2011	5	27	9	27	1.9%	0.56 [0.21, 1.44]
Luo SW 2007	3	30	7	30	1.2%	0.43 [0.12, 1.50]
Luo SZ 2006	4	25	14	25	1.9%	0.29 [0.11, 0.75]
Wang LY 2009	12	40	22	40	4.7%	0.55 [0.31, 0.95]
Zhang FL 2008	4	30	8	30	1.5%	0.50 [0.17, 1.48]
**Subtotal (95% CI)**		**202**		**192**	**15.8%**	**0.45 [0.33, 0.62]**
Total events	40		84			
Heterogeneity: Tau² = 0.00; Chi² = 1.73, df = 5 (P = 0.88); I² = 0%						
Test for overall effect: Z = 4.97 (P < 0.00001)						
**2.8.4 SFI+DP/DP**						
Ma CG 2013	8	28	16	28	3.5%	0.50 [0.26, 0.97]
Yu F 2007	10	30	11	30	3.3%	0.91 [0.46, 1.81]
**Subtotal (95% CI)**		**58**		**58**	**6.8%**	**0.67 [0.37, 1.20]**
Total events	18		27			
Heterogeneity: Tau² = 0.06; Chi² = 1.49, df = 1 (P = 0.22); I² = 33%						
Test for overall effect: Z = 1.34 (P = 0.18)						
**2.8.5 SFI+NC/NC**						
Jia YL 2012	28	72	42	71	8.4%	0.66 [0.46, 0.93]
Wang YZ 2007	4	28	5	27	1.2%	0.77 [0.23, 2.57]
**Subtotal (95% CI)**		**100**		**98**	**9.6%**	**0.67 [0.48, 0.93]**
Total events	32		47			
Heterogeneity: Tau² = 0.00; Chi² = 0.06, df = 1 (P = 0.80); I² = 0%						
Test for overall effect: Z = 2.39 (P = 0.02)						
**Total (95% CI)**		**1027**		**1017**	**100.0%**	**0.62 [0.54, 0.72]**
Total events	280		453			
Heterogeneity: Tau² = 0.03; Chi² = 32.52, df = 25 (P = 0.14); I² = 23%						
Test for overall effect: Z = 6.65 (P < 0.00001)						
Test for subgroup differences: Chi² = 4.60, df = 4 (P = 0.33), I² = 13.0%						

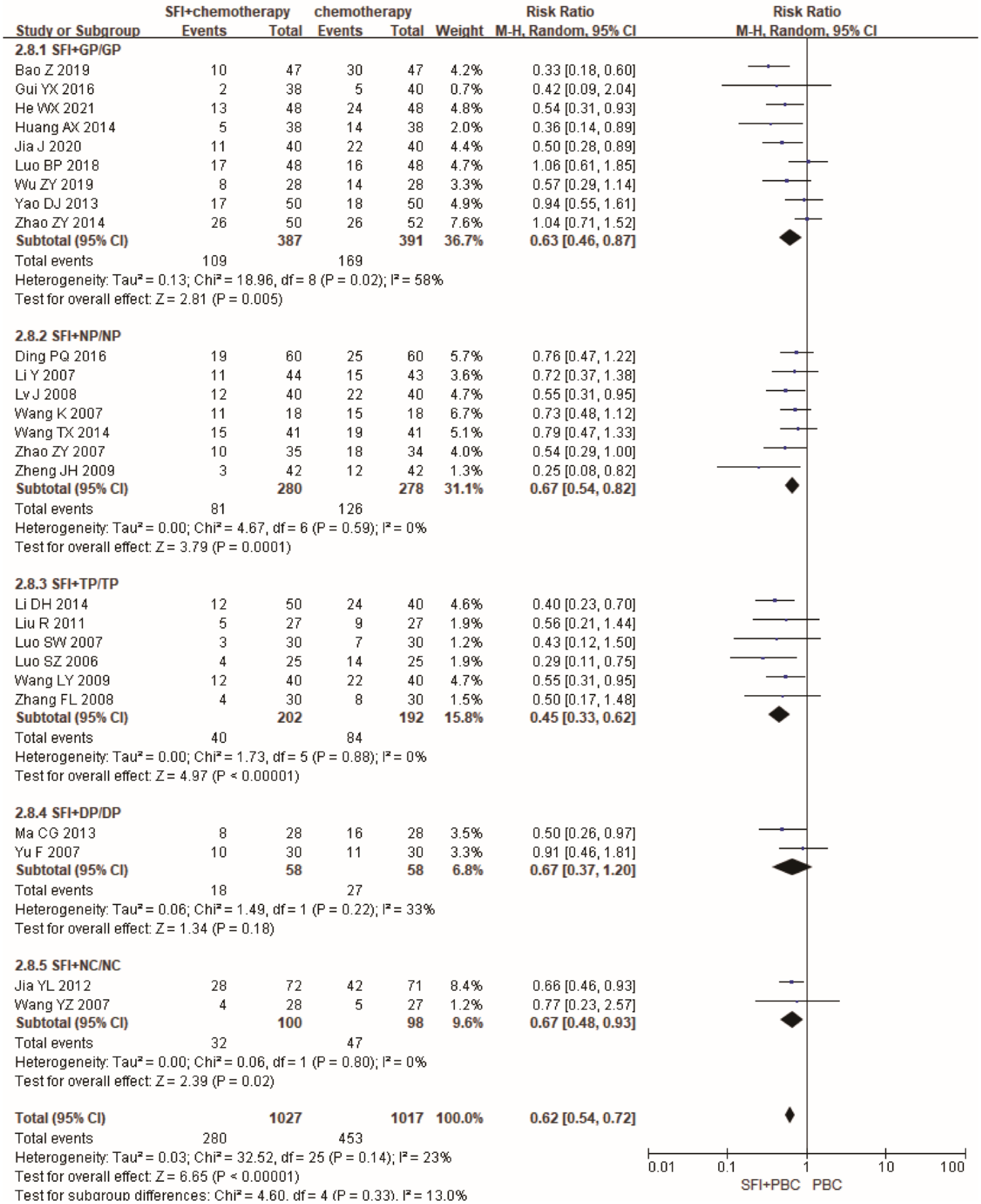

Supplementary Figure 6 Forest plot of the incidence of thrombocytopenia stratified by chemotherapy regimen

Note: GP,gemcitabine + cisplatin; NP,vinorelbine + cisplatin; TP,paclitaxel / albumin paclitaxel / paclitaxel liposome + cisplatin; DP,docetaxel + cisplatin ; NC,vinorelbine + carboplatin.

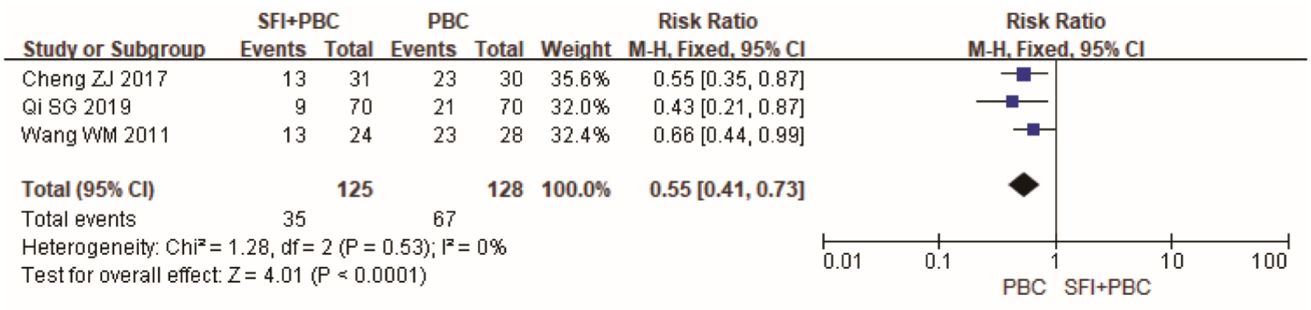

Supplementary Figure 7 Forest plot of incidence of myelosuppression alone

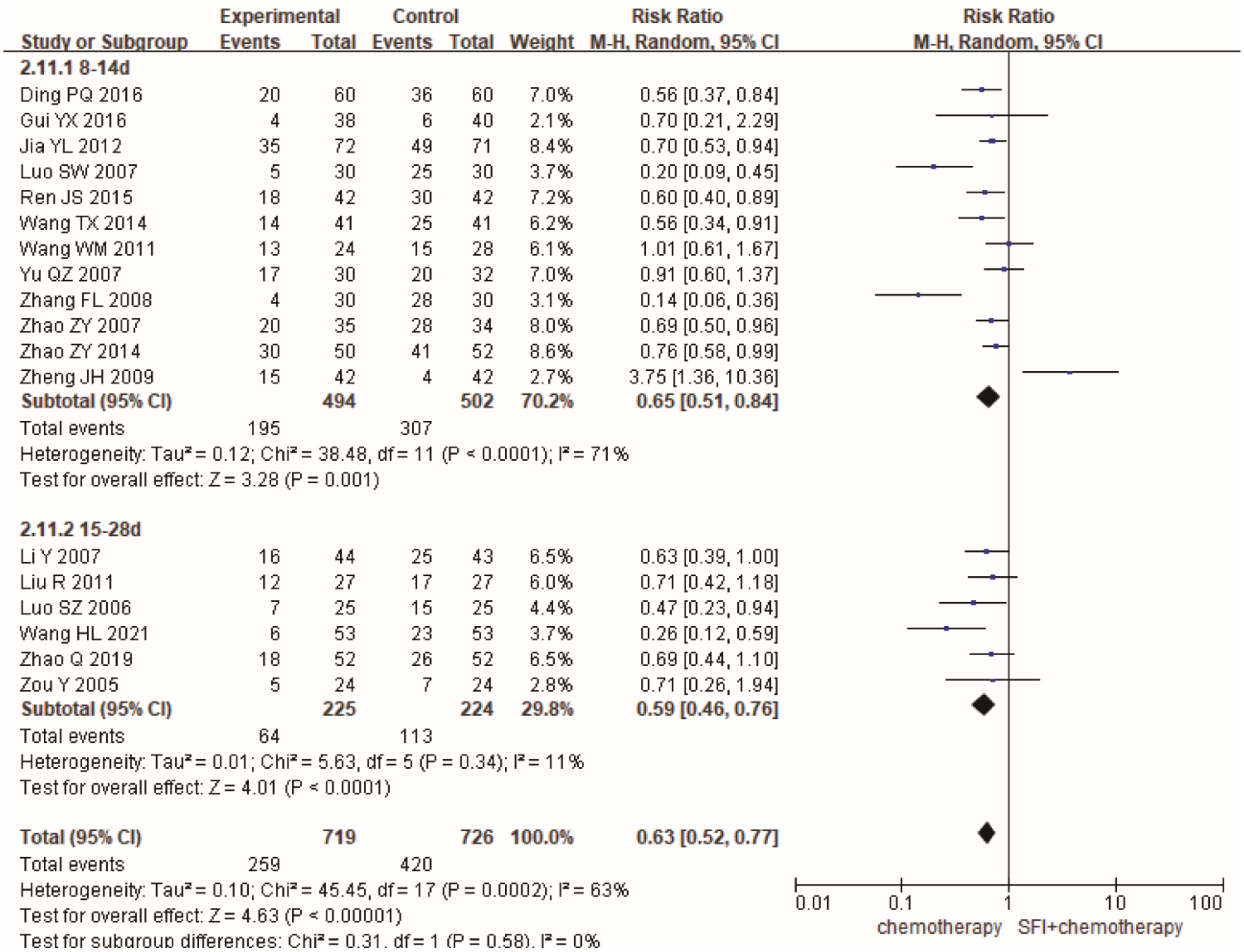

Supplementary Figure 8 Forest plot of the incidence of nausea and vomiting stratified by days of single cycle SFI dosing

Study or Subgroup	SFI+chemotherapy Events	SFI+chemotherapy Total	chemotherapy Events	chemotherapy Total	Weight	Risk Ratio M-H, Random, 95% CI
**2.12.1 SFI+GP/GP**						
Gui YX 2016	4	38	6	40	2.1%	0.70 [0.21, 2.29]
Wang HL 2021	6	53	23	53	3.7%	0.26 [0.12, 0.59]
Zhao ZY 2014	30	50	41	52	8.6%	0.76 [0.58, 0.99]
**Subtotal (95% CI)**		**141**		**145**	**14.4%**	**0.53 [0.24, 1.19]**
Total events	40		70			
Heterogeneity: Tau² = 0.36; Chi² = 7.47, df = 2 (P = 0.02); I² = 73%						
Test for overall effect: Z = 1.54 (P = 0.12)						
**2.12.2 SFI+NP/NP**						
Ding PQ 2016	20	60	36	60	7.0%	0.56 [0.37, 0.84]
Li Y 2007	16	44	25	43	6.5%	0.63 [0.39, 1.00]
Wang TX 2014	14	41	25	41	6.2%	0.56 [0.34, 0.91]
Yu QZ 2007	17	30	20	32	7.0%	0.91 [0.60, 1.37]
Zhao ZY 2007	20	35	28	34	8.0%	0.69 [0.50, 0.96]
Zheng JH 2009	15	42	4	42	2.7%	3.75 [1.36, 10.36]
**Subtotal (95% CI)**		**252**		**252**	**37.4%**	**0.75 [0.54, 1.04]**
Total events	102		138			
Heterogeneity: Tau² = 0.10; Chi² = 14.45, df = 5 (P = 0.01); I² = 65%						
Test for overall effect: Z = 1.74 (P = 0.08)						
**2.12.3 SFI+TP/TP**						
Liu R 2011	12	27	17	27	6.0%	0.71 [0.42, 1.18]
Luo SW 2007	5	30	25	30	3.7%	0.20 [0.09, 0.45]
Luo SZ 2006	7	25	15	25	4.4%	0.47 [0.23, 0.94]
Zhang FL 2008	4	30	28	30	3.1%	0.14 [0.06, 0.36]
Zhao Q 2019	18	52	26	52	6.5%	0.69 [0.44, 1.10]
**Subtotal (95% CI)**		**164**		**164**	**23.7%**	**0.40 [0.22, 0.72]**
Total events	46		111			
Heterogeneity: Tau² = 0.35; Chi² = 17.26, df = 4 (P = 0.002); I² = 77%						
Test for overall effect: Z = 3.02 (P = 0.003)						
**2.12.4 SFI+DP/DP**						
Wang WM 2011	13	24	15	28	6.1%	1.01 [0.61, 1.67]
**Subtotal (95% CI)**		**24**		**28**	**6.1%**	**1.01 [0.61, 1.67]**
Total events	13		15			
Heterogeneity: Not applicable						
Test for overall effect: Z = 0.04 (P = 0.97)						
**2.12.5 SFI+NC/NC**						
Jia YL 2012	35	72	49	71	8.4%	0.70 [0.53, 0.94]
**Subtotal (95% CI)**		**72**		**71**	**8.4%**	**0.70 [0.53, 0.94]**
Total events	35		49			
Heterogeneity: Not applicable						
Test for overall effect: Z = 2.42 (P = 0.02)						
**2.12.6 SFI+AP/AP**						
Ren JS 2015	18	42	30	42	7.2%	0.60 [0.40, 0.89]
**Subtotal (95% CI)**		**42**		**42**	**7.2%**	**0.60 [0.40, 0.89]**
Total events	18		30			
Heterogeneity: Not applicable						
Test for overall effect: Z = 2.51 (P = 0.01)						
**2.12.7 SFI+TC/TC**						
Zou Y 2005	5	24	7	24	2.8%	0.71 [0.26, 1.94]
**Subtotal (95% CI)**		**24**		**24**	**2.8%**	**0.71 [0.26, 1.94]**
Total events	5		7			
Heterogeneity: Not applicable						
Test for overall effect: Z = 0.66 (P = 0.51)						
**Total (95% CI)**		**719**		**726**	**100.0%**	**0.63 [0.52, 0.77]**
Total events	259		420			
Heterogeneity: Tau² = 0.10; Chi² = 45.45, df = 17 (P = 0.0002); I² = 63%						
Test for overall effect: Z = 4.63 (P < 0.00001)						
Test for subgroup differences: Chi² = 6.63, df = 6 (P = 0.36), I² = 9.5%						

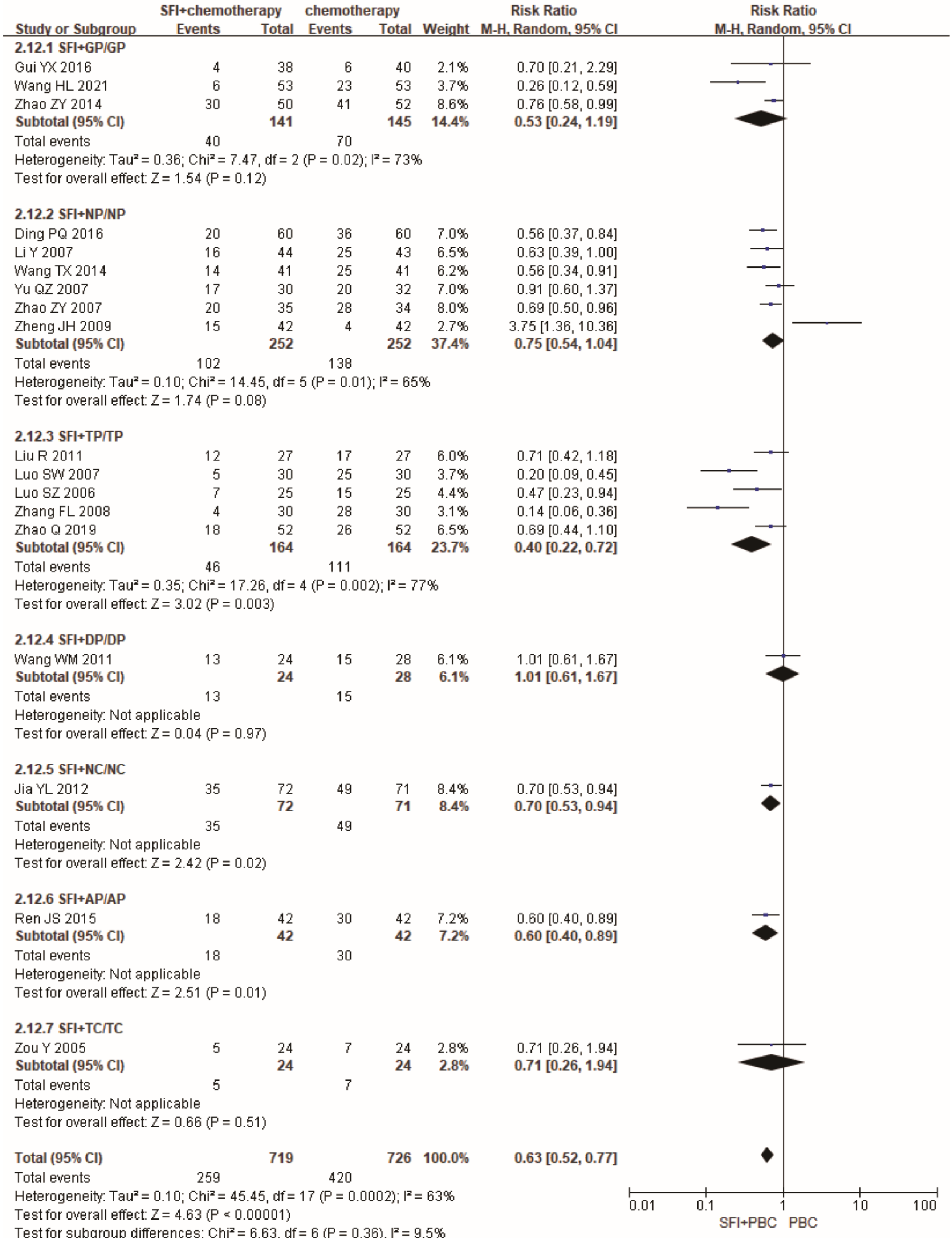

Supplementary Figure 9 Forest plot of incidence of nausea and vomiting stratified by chemotherapy regimen

Note: GP,gemcitabine + cisplatin; NP,vinorelbine + cisplatin; TP,paclitaxel / albumin paclitaxel / paclitaxel liposome + cisplatin; DP,docetaxel + cisplatin ; NC,vinorelbine + carboplatin; AP,pemetrexed + cisplatin; TC,paclitaxel / albumin paclitaxel / paclitaxel liposome + carboplatin.

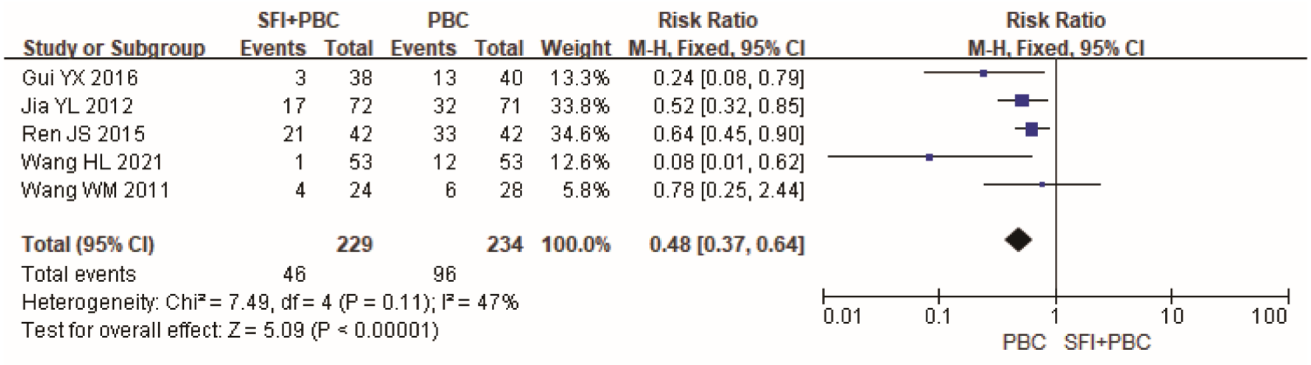

Supplementary Figure 10 Forest plot of incidence of diarrhea

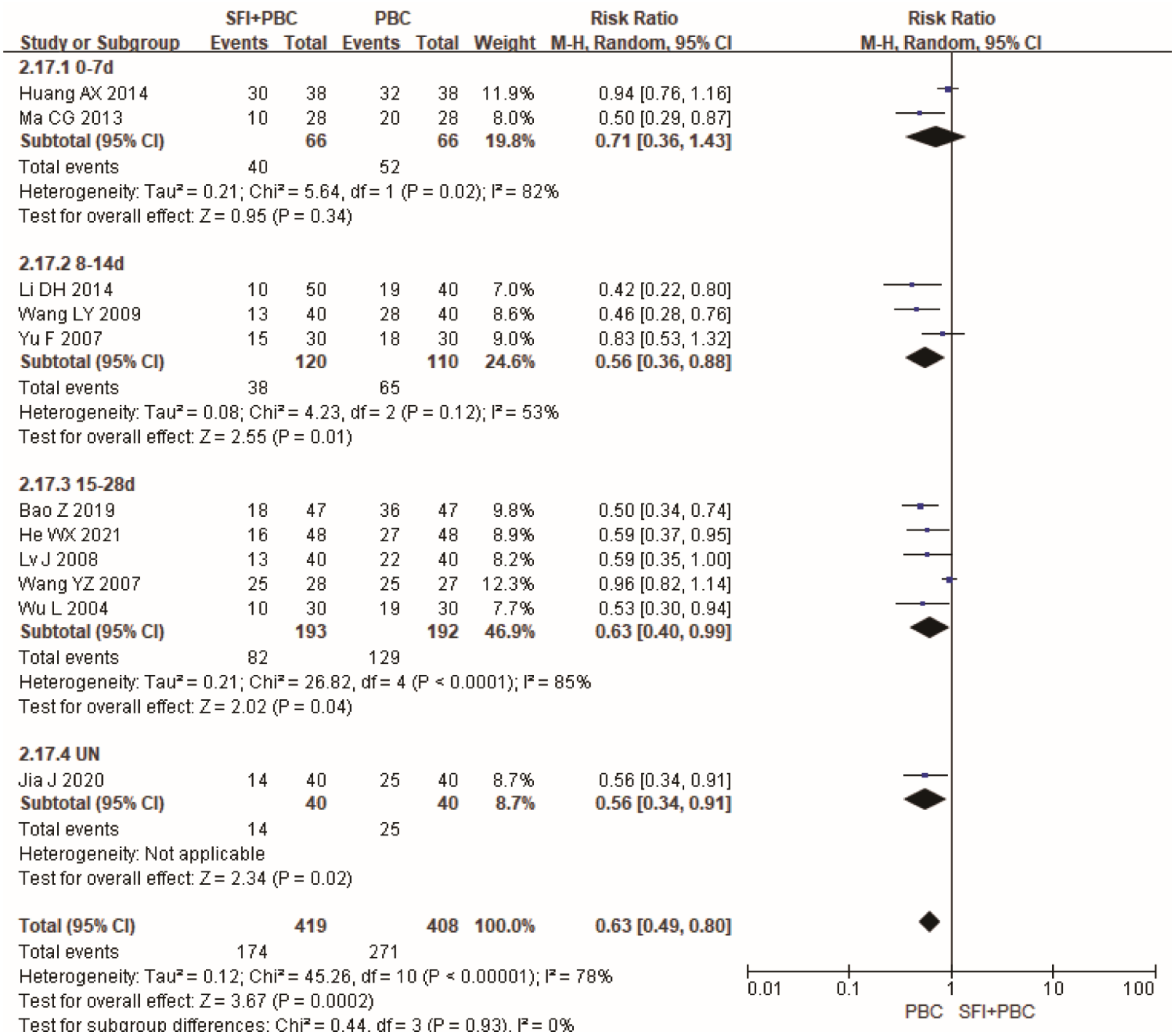

Supplementary Figure 11 Forest plot of incidence of simple gastrointestinal reactions stratified by days of single-cycle SFI dosing

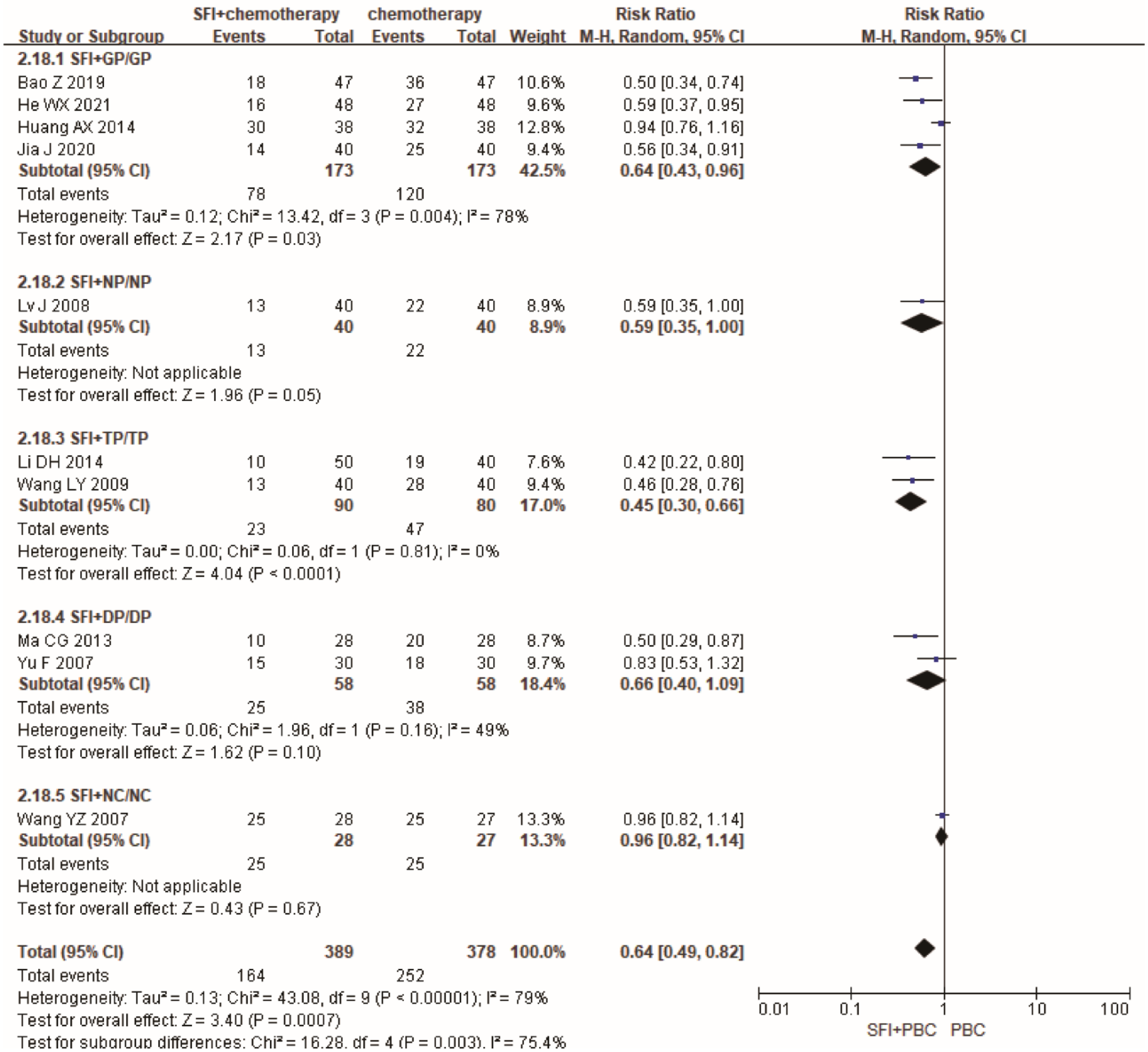

Supplementary Figure 12 Forest plot of incidence of GI-only reactions stratified by chemotherapy regimen

Note: GP,gemcitabine + cisplatin;NP,vinorelbine + cisplatin; TP,paclitaxel / albumin paclitaxel / paclitaxel liposome + cisplatin; DP,docetaxel + cisplatin; NC,vinorelbine + carboplatin.
